# Asymmetric catalysis mediated by a mirror symmetry-broken helical nanoribbon

**DOI:** 10.1038/s41467-019-11840-3

**Published:** 2019-09-04

**Authors:** Zhaocun Shen, Yutao Sang, Tianyu Wang, Jian Jiang, Yan Meng, Yuqian Jiang, Kou Okuro, Takuzo Aida, Minghua Liu

**Affiliations:** 1 Beijing National Laboratory for Molecular Sciences, CAS Key Laboratory of Colloid, Interface and Chemical Thermodynamics, CAS Research/Education Center for Excellence in Molecular Sciences, Institute of Chemistry, Chinese Academy of Sciences, Beijing, 100190 P. R. China; 2 Department of Chemistry and Biotechnology, School of Engineering, The University of Tokyo, 7-3-1 Hongo, Bunkyo-ku, Tokyo, 113-8656 Japan; 3 University of Chinese Academy of Sciences, Beijing, 100049 P. R. China; 4 CAS Center for Excellence in Nanoscience, National Center for Nanoscience and Technology, Beijing, 100190 P. R. China; 5 RIKEN Center for Emergent Matter Science, 2-1 Hirosawa, Wako, Saitama, 351-0198 Japan; 6 Collaborative Innovation Center of Chemical Science and Engineering, Tianjin, 300072 P. R. China

**Keywords:** Asymmetric catalysis, Organocatalysis, Self-assembly

## Abstract

Although chirality has been recognized as an essential entity for life, it still remains a big mystery how the homochirality in nature emerged in essential biomolecules. Certain achiral motifs are known to assemble into chiral nanostructures. In rare cases, their absolute geometries are enantiomerically biased by mirror symmetry breaking. Here we report the first example of asymmetric catalysis by using a mirror symmetry-broken helical nanoribbon as the ligand. We obtain this helical nanoribbon from a benzoic acid appended achiral benzene-1,3,5-tricarboxamide by its helical supramolecular assembly and employ it for the Cu^2+^-catalyzed Diels–Alder reaction. By thorough optimization of the reaction (conversion: > 99%, turnover number: ~90), the enantiomeric excess eventually reaches 46% (major/minor enantiomers = 73/27). We also confirm that the helical nanoribbon indeed carries helically twisted binding sites for Cu^2+^. Our achievement may provide the fundamental breakthrough for producing optically active molecules from a mixture of totally achiral motifs.

## Abstract (web summary)

If asymmetric catalysts were available by mirror symmetry breaking, an important insight may be given to how the biomolecular homochirality emerged in nature. Here, the authors report the first example of asymmetric catalysis by employing mirror symmetry-broken helical nanoribbons as the ligand.

## Introduction

Chirality has been recognized as an essential entity for life. However, it still remains a big mystery how the homochirality in nature emerged in essential biomolecules, such as sugars and amino acids^1,2^. Certain achiral molecules are known to form crystals adopting a chiral geometry, which usually form as a conglomerate, i.e., a racemic mixture of enantiomorphic crystals. Post chemical transformation of such geometrically chiral single crystals may afford enantiomerically enriched products^3–5^. This is one of the typical approaches to absolute asymmetric synthesis^6–8^, which has attracted long-term attention since the time of Pasteur. If mirror symmetry breaking occurs in the crystallization of achiral compounds, a mixture of unequal numbers of enantiomorphic crystals forms. For example, Kondepudi et al. reported that stirred crystallization is often effective to induce mirror symmetry breaking, leading to a high enantiomeric excess (*ee*) of the resulting crystal^9–11^. Notably, Viedma et al. later demonstrated that continuous grinding of a slurry of a racemic mixture of enantiomorphic crystals could cause its deracemization to produce an enantiopure product (Viedma ripening)^12–18^. Especially, Blackmond and coworkers for the first time experimentally supported a hypothesis of the emergence of solid-phase homochirality from racemic conglomerates composed of rapidly racemizing enantiomers^13,19,20^. Tsogoeva and coworkers achieved a complete deracemization (100% *ee*) of an asymmetric reaction product by combining its reversible asymmetric reaction and physical grinding of crystals involved^21,22^. Nevertheless, an even more challenging subject to tackle is how one can amplify the enantiomeric bias generated as a consequence of mirror symmetry breaking. In 2008, Soai and coworkers employed such a mirror symmetry-broken crystalline mixture of cytosine as a chiral auxiliary for the enantioselective addition reaction between diisopropylzinc and pyrimidine-5-carbaldehyde^23^. Although the range of eligible substrates is highly limited, this reaction is well-known for amplifying the enantiomeric bias in an autocatalytic manner^24^.

Some covalent helical polymers^25–35^ and their noncovalent analogues^36–40^, prepared from certain chiral monomers, are known to serve as chiral ligands for asymmetric catalysis. Suginome and coworkers have developed a series of helical poly(quinoxaline-2,3-diyl) copolymers bearing metal-binding phosphine pendants as chiral ligands for several palladium-catalyzed asymmetric reactions^29–34^. Examples of noncovalent helical supramolecular assemblies include hydrogen-bonded helical stacks of chiral benzene-1,3,5-tricarboxamide (BTA) derivatives^37–40^. In our previous paper, we reported that an achiral BTA motif that was appended with an ethyl cinnamate group in each of its three side chains self-assembled into helical nanoribbons, where mirror symmetry breaking occurred under certain conditions to form a mixture of unequal numbers of right-handed (*P*) and left-handed (*M*) helical nanoribbons^41,42^. We envisioned that such mirror symmetry-broken supramolecular assemblies^41–54^ might mediate asymmetric reactions if they are properly designed to accommodate catalytically active transition metal ions.

**Fig. 1:**
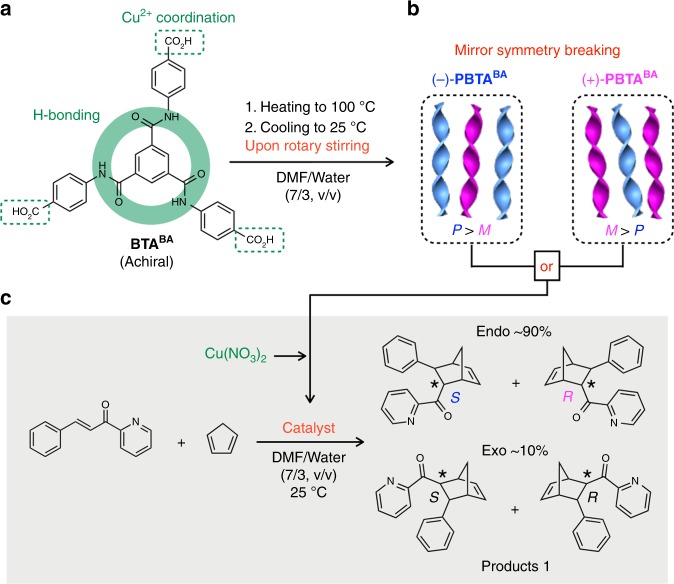
Mirror symmetry breaking of helical supramolecular nanoribbons for asymmetric catalysis. **a**, **b** Molecular structure of the achiral **BTA**^**BA**^ monomer and schematic representation of the preparation of helical **PBTA**^**BA**^ nanoribbons with either (*P*)- or (*M*)-dominant helical handedness by applying rotary stirring during the cooling process of a DMF/water solution of **BTA**^**BA**^. (*P*)- and (*M*)-dominant **PBTA**^**BA**^ display negative ((−)-**PBTA**^**BA**^) and positive ((+)-**PBTA**^**BA**^) CD signs at 316 nm, respectively. **c** Diels–Alder cycloaddition reaction between aza-chalcone and cyclopentadiene catalyzed by Cu^2+^ in combination with (−)-**PBTA**^**BA**^ or (+)-**PBTA**^**BA**^. The reaction affords the endo isomer preferentially (~90%). The (*S*)-endo and (*R*)-endo enantiomers were obtained preferentially when (−)-**PBTA**^**BA**^ and (+)-**PBTA**^**BA**^ were used, respectively

Herein, we report the first example of asymmetric catalysis realized by using a mirror symmetry-broken helical supramolecular assembly of an achiral BTA motif as the ligand (Fig. 1). The achiral BTA derivative (**BTA**^**BA**^, Fig. 1a)^55^ carries a benzoic acid group in each of its three arms for chelating a Cu^2+^ ion that can catalyze the Diels–Alder cycloaddition reaction between aza-chalcone and cyclopentadiene (Fig. 1c). The enantiomeric excess of the reaction reaches 46% (conversion: >99%, turnover number: ~90).

## Results

### Mirror symmetry breaking of helical nanoribbons

**Fig. 2:**
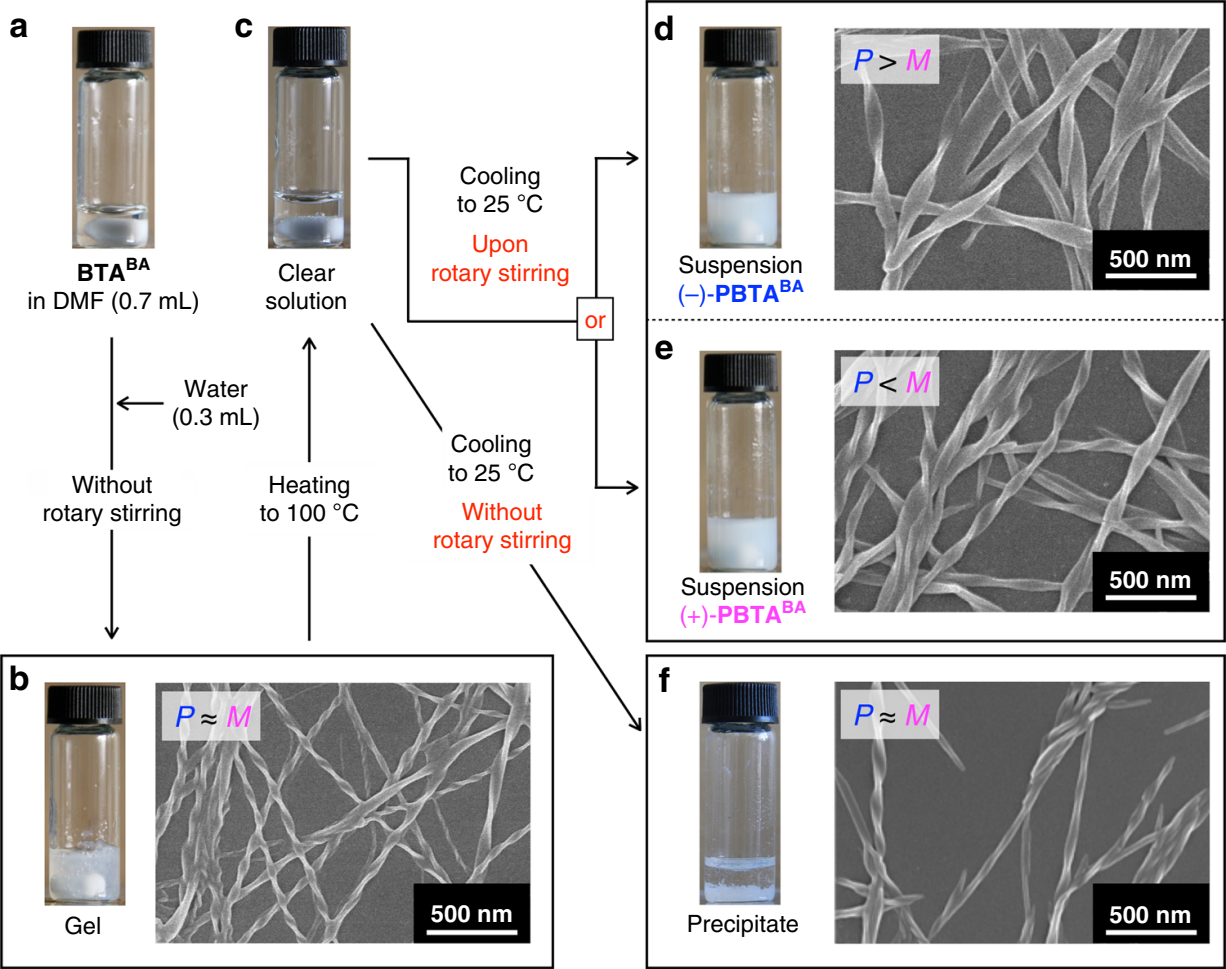
Procedures for the preparation of mirror symmetry-broken helical **PBTA**^**BA**^ nanoribbons. **a** Photograph of a DMF (0.7 mL) solution of **BTA**^**BA**^ (3 mg, 5.3 × 10^−6^ mol). **b** Photograph of a CD-silent gel of **PBTA**^**BA**^ ([**BTA**^**BA**^] = 5.3 mM) formed in DMF/water (7/3, v/v; 1 mL) and SEM image of its air-dried specimen. The gel can be prepared via an antisolvent method through direct addition of water (poor solvent) into a DMF (good solvent) solution of **BTA**^**BA**^ without magnetic rotary stirring. **c** Photograph of a DMF/water (7/3, v/v; 1 mL) solution of **BTA**^**BA**^ (5.3 mM) after heating at 100 °C for 3 min. **d**, **e** Typical photographs of CD-active DMF/water (7/3, v/v; 1 mL) suspensions of **PBTA**^**BA**^ ([**BTA**^**BA**^] = 5.3 mM) and SEM images of their air-dried specimens. The suspensions of (−)-**PBTA**^**BA**^ and (+)-**PBTA**^**BA**^ display mirror-image CD spectra of one another (Fig. 4b). They can be prepared by natural cooling of a clear, hot DMF/water (7/3, v/v; 1 mL) solution of **BTA**^**BA**^ (5.3 mM) with magnetic rotary stirring at 1200 rpm for 40 min. **f** Photograph of a CD-silent precipitate of **PBTA**^**BA**^ ([**BTA**^**BA**^] = 5.3 mM) in DMF/water (7/3, v/v; 1 mL) and SEM image of its air-dried specimen. This precipitate can be prepared by natural cooling of a clear, hot DMF/water (7/3, v/v; 1 mL) solution of **BTA**^**BA**^ (5.3 mM) without magnetic rotary stirring

**BTA**^**BA**^ was synthesized and unambiguously characterized (see Supplementary Methods, Supplementary Figs. 1−6). Analogous to previous examples^41,42,55^, **BTA**^**BA**^ self-assembled into **PBTA**^**BA**^, a supramolecular helical nanoribbon (Fig. 1b). We found that this self-assembly event proceeds with mirror symmetry breaking to afford either (*P*)-dominant **PBTA**^**BA**^ ((−)-**PBTA**^**BA**^) or (*M*)-dominant **PBTA**^**BA**^ ((**+**)-**PBTA**^**BA**^) (Fig. 1b) under physical agitation generated by magnetic rotary stirring. The method for the preparation of mirror symmetry-broken **PBTA**^**BA**^ is slightly complicated as described below (Fig. 2) but quite analogous to that reported in our previous paper^42^: Water (0.3 mL) was added to a *N,N*-dimethylformamide (DMF, 0.7 mL) solution of **BTA**^**BA**^ (3 mg, 5.3 × 10^−6^ mol) in a 5-mL cylindrical glass vial (Fig. 2a), whereupon the mixture, when allowed to stand at 25 °C for 2 h, underwent physical gelation (Fig. 2b and Supplementary Tables 1 and 2). The gel was heated at 100 °C for 3 min, and the resultant homogeneous solution (Fig. 2c) was allowed to cool to 25 °C with magnetic rotary stirring (1200 rpm) for 40 min, yielding a white suspension (Fig. 2d, e), which turned out to be optically active (see below). This result was highly reproducible (Supplementary Fig. 7), except that the sign of the circular dichroism (CD) activity at 316 nm of the resultant suspension was either negative ((−)-**PBTA**^**BA**^) or positive ((**+**)-**PBTA**^**BA**^) stochastically (see below). Although the CD sign was independent of the direction of rotary stirring (Supplementary Fig. 7), a stable CD activity, once emerged, lasted without fading unless the suspension was heated to transform into a homogeneous solution. Note that, as demonstrated for the suspension of (−)-**PBTA**^**BA**^ in Supplementary Fig. 8, its CD spectrum was barely contaminated with a spectral artifact due to linear dichroism (LD). Next, an aliquot of the suspension was taken out from the glass vial and cast on a silicon wafer for air-drying, followed by evacuation under reduced pressure. Scanning electron microscopy (SEM) of the resulting specimens revealed that (−)-**PBTA**^**BA**^ consisted mostly of (*P*)-helical nanoribbons (Fig. 2d), whereas (*M*)-helical nanoribbons were dominant in (**+**)-**PBTA**^**BA**^ (Fig. 2e). The gel (Fig. 2b) was CD-silent (Supplementary Figs. 9 and 10). Consistently, its cross-linked 3D network appeared to be composed of equal numbers of (*P*)- and (*M*)-helical nanoribbons (Fig. 2b). The same held true when a clear, hot solution of **BTA**^**BA**^ (Fig. 2c) was allowed to cool to 25 °C without rotary stirring (Fig. 2f and Supplementary Fig. 11). Not only magnetic rotary stirring but also mechanical rotary stirring using an overhead stirrer, vortex mixing, and even sonication resulted in the formation of a CD-active suspension of (−)-**PBTA**^**BA**^ or (+)-**PBTA**^**BA**^ (Supplementary Fig. 12).

Interestingly, an ethyl ester derivative of **BTA**^**BA**^ did not assemble into helical nanoribbons under otherwise identical conditions to those for **BTA**^**BA**^ (Supplementary Fig. 13), suggesting that the peripheral carboxylic acid groups of **BTA**^**BA**^ play a critical role in its helical assembly. Fourier transform infrared (FT-IR) spectroscopy of a xerogel of **PBTA**^**BA**^, dried under reduced pressure, showed small vibrational bands at 2665 and 2543 cm^−1^ (Supplementary Fig. 14), which are characteristic of the OH stretching vibrations due to hydrogen-bonded dimers of carboxylic acids. Two bands at 3311 and 1654 cm^−1^, assignable to the stretching vibrations of hydrogen-bonded amide NH and C = O groups, respectively, were also observed. We found that **BTA**^**BA**^ is quite soluble in DMF alone ([**BTA**^**BA**^] = 5.3 mM), where it showed a much sharper absorption band at 290 nm (Supplementary Fig. 15) than its self-assembled state ([**BTA**^**BA**^] =5.3 mM) in the gel formed in DMF/water (7/3, v/v, Fig. 2b). Compared with the DMF solution of **BTA**^**BA**^, the gel was much more luminescent (~300 times) at 447 nm upon excitation at 290 nm (Supplementary Fig. 16), suggesting that the **BTA**^**BA**^ units in the gel network are located in a highly congested environment. Further to note, a DMF/water suspension of **PBTA**^**BA**^ (Fig. 2d/e) was silent in polarized optical microscopy (Supplementary Fig. 17), and a wet sample of **PBTA**^**BA**^ isolated by centrifugation of its suspension showed no X-ray diffraction (Supplementary Fig. 18). These results allow us to conclude that **PBTA**^**BA**^ is a noncrystalline material.

When a clear, hot solution of **BTA**^**BA**^ (Fig. 2c) in DMF/water (7/3, v/v) was allowed to cool to 25 °C with continuous rotary stirring, the absorption dissymmetry factor (*g*_abs_) of the resulting suspension, evaluated from the CD intensity relative to the absorbance at 316 nm, gradually increased with the stirring time and then leveled off in 40 min at 2.2 × 10^−2^ (Supplementary Fig. 19). We noticed that faster stirring resulted in the emergence of a larger CD intensity at 316 nm (Supplementary Fig. 20). Rotary stirring of the CD-silent gel did not induce any CD band (Supplementary Fig. 21), indicating that physical agitation such as rotary stirring does not affect the helical handedness of the **PBTA**^**BA**^ nanoribbons once formed. From these observations, we proposed a mechanism for the formation of the (*P*)- or (*M*)-dominant helical nanoribbons from **BTA**^**BA**^ as follows: **BTA**^**BA**^ self-assembles into (*P*)- and (*M*)-helical short 1D aggregates (primary nucleation). Such aggregates grow up to longer (*P*)- and (*M*)-helical nanoribbons, respectively. By physical agitation, the long helical nanoribbons preferentially break up into numerous short aggregates with the same handedness (secondary nucleation). In the resulting nonequilibrated state, once the mirror symmetry of the **PBTA**^**BA**^ nanoribbon is broken as a consequence of stochastic fluctuation, a small chiral bias thus generated can be amplified through repeated cycles of the secondary nucleation, as proposed for the mechanism of chiral amplification via thermal cycling or attrition in conglomerate crystal systems^9–11^. Accordingly, the addition of (*P*)-dominant **PBTA**^**BA**^ as a seed into a DMF/water (7/3, v/v; 1 mL) solution of **BTA**^**BA**^ (5.3 mM) before rotary stirring always resulted in the formation of a suspension of (*P*)-dominant **PBTA**^**BA**^ with a negative CD band at 316 nm (Supplementary Fig. 22).

### **A**symmetric catalysis

**Fig. 3:**
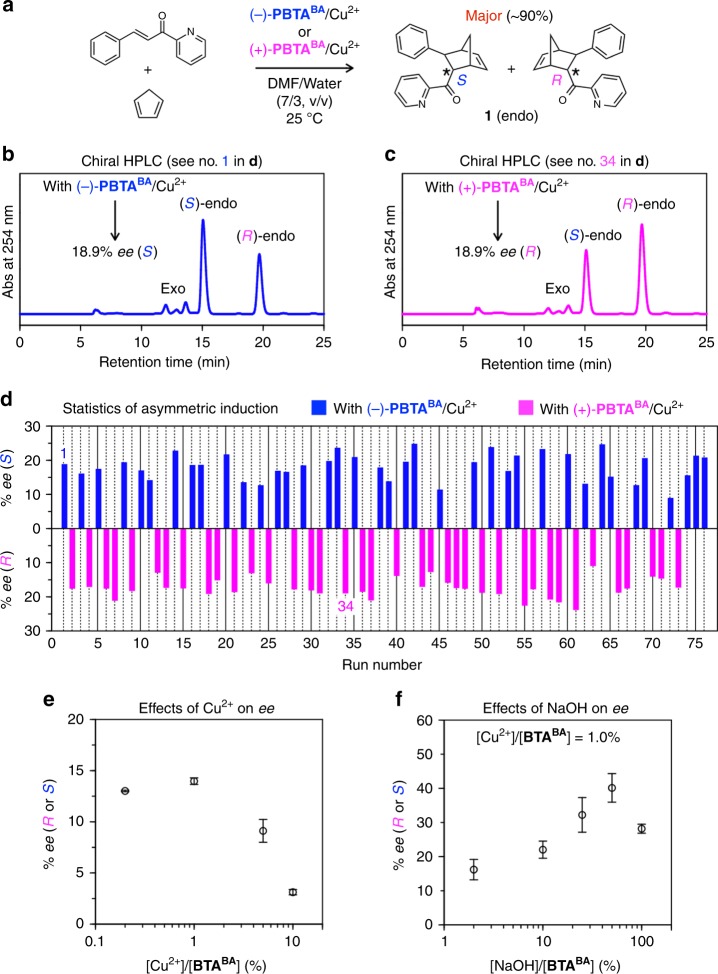
Enantioselective Diels–Alder reaction catalyzed by Cu^2+^-coordinated **PBTA**^**BA**^ nanoribbons. **a** Diels–Alder reaction between aza-chalcone and cyclopentadiene catalyzed by (−)-**PBTA**^**BA**^/Cu^2+^ or (+)-**PBTA**^**BA**^/Cu^2+^, which preferentially affords the endo isomer (~90%). **b**, **c** Chiral HPLC traces of the reaction mixtures in run No. 1 (**b**) and 34 (**c**) in (**d**). Symbols *ee* (*S*) and *ee* (*R*) represent enantiomeric excess (*ee*) values of the endo isomers with the dominancy of their (*S*)-endo and (*R*)-endo enantiomers, respectively. **d** Statistical *ee* values of the endo isomers obtained from a total of 76 runs of the Diels–Alder reaction catalyzed at 25 °C by 76 different batches of (–)-**PBTA**^**BA**^/Cu^2+^ and (+)-**PBTA**^**BA**^/Cu^2+^ at [Cu^2+^]/[**BTA**^**BA**^] = 1.0%. Blue and pink-colored bars represent the *ee* values of the endo isomers obtained when (−)-**PBTA**^**BA**^/Cu^2+^ and (+)-**PBTA**^**BA**^/Cu^2+^, respectively, are used as the catalysts. **e** Effects of [Cu^2+^]/[**BTA**^**BA**^] on the *ee* values of the endo isomers obtained at 25 °C with (−)-**PBTA**^**BA**^/Cu^2+^ or (+)-**PBTA**^**BA**^/Cu^2+^. **f** Effects of [NaOH]/[**BTA**^**BA**^] on the *ee* values of the endo isomers obtained at 25 °C with (−)-**PBTA**^**BA**^/Cu^2+^ or (+)-**PBTA**^**BA**^/Cu^2+^ at [Cu^2+^]/[**BTA**^**BA**^] = 1.0%, where NaOH was added to the suspensions of (−)-**PBTA**^**BA**^ or (+)-**PBTA**^**BA**^ prior to the addition of Cu(NO_3_)_2_. Error bars represent the standard deviation. Source data are provided as a Source Data file

Previously, groups of Engberts^56,57^ and Roelfes^25,27^ reported highly enantioselective Cu^2+^-catalyzed Diels–Alder reactions between aza-chalcone and cyclopentadiene using amino acids and DNA as the chiral mediators, respectively. Inspired by these successful examples, we chose the same reaction to investigate a possibility of mirror symmetry-broken **PBTA**^**BA**^ nanoribbons as a chiral ligand. Typically, Cu^2+^-coordinated (−)-**PBTA**^**BA**^ or (+)-**PBTA**^**BA**^ ([Cu^2+^]/[**BTA**^**BA**^] = 1.0%), employed as a catalyst for the Diels–Alder reaction (Fig. 3a), was prepared by mixing an aqueous solution of Cu(NO_3_)_2_ (4.1 mM, 13 µL) and a DMF/water (7/3, v/v; 1 mL) suspension of (−)-**PBTA**^**BA**^ or (+)-**PBTA**^**BA**^ ([**BTA**^**BA**^] = 5.3 mM, Fig. 2d, e) at 25 °C for 1 h. The luminescence of **PBTA**^**BA**^ was quenched upon being mixed with Cu(NO_3_)_2_ (Supplementary Fig. 23), suggesting that **PBTA**^**BA**^ accommodates Cu^2+^ presumably at the peripheral carboxylate units. As observed by SEM and CD spectroscopy, **PBTA**^**BA**^ maintained its helical nanoribbon structure even after being mixed with Cu^2+^ (Supplementary Fig. 24). For the Diels–Alder reaction, an acetonitrile (40 µL) solution of aza-chalcone (1 mg) and freshly distilled cyclopentadiene (40 µL) were successively added to a DMF/water (7/3, v/v) suspension of (−)-**PBTA**^**BA**^/Cu^2+^ or (+)-**PBTA**^**BA**^/Cu^2+^, and the mixture was stirred at 25 °C. After 36 h, the reaction mixture was extracted with ethyl acetate (0.5 mL), and an organic extract, after being evaporated to dryness, was subjected to chiral high performance liquid chromatography (HPLC), where the endo isomer was revealed to be the major product (∼90%, conversion: >99%, turnover number: ~90, Fig. 3b, c). Of particular interest, (−)-**PBTA**^**BA**^/Cu^2+^ (Fig. 2d) and (+)-**PBTA**^**BA**^/Cu^2+^ (Fig. 2e) preferentially afforded the enantiomers of (*S*)-endo (Fig. 3b) and (*R*)-endo (Fig. 3c), respectively. We conducted a total of 76 runs of the Diels–Alder reaction using 76 different batches of (−)-**PBTA**^**BA**^/Cu^2+^ and (+)-**PBTA**^**BA**^/Cu^2+^ under the conditions described above and confirmed that this stereochemical correlation between the catalyst and product was perfectly reproduced (Fig. 3d and Supplementary Table 3). However, there is no clear correlation between the CD intensities of the catalyst and enantiomeric excess values of the product (Supplementary Fig. 25). We confirmed that the nanoribbons of **PBTA**^**BA**^ after the reaction were shorter than those before the reaction (Supplementary Fig. 26). However, the *ee* value of the endo isomer did not substantially change with the reaction time (Supplementary Fig. 27). In sharp contrast, repeated usage of the catalyst, isolated each time via centrifugation from the reaction mixture, resulted in lowering of the enantiomeric excess of the product (Supplementary Table 4). Meanwhile, CD-silent **PBTA**^**BA**^/Cu^2+^ nanoribbons (Fig. 2b) also catalyzed the Diels–Alder reaction. However, as expected, the reaction was not enantioselective (Supplementary Fig. 28), as in the case where **PBTA**^**BA**^ was not employed for the Cu^2+^-catalyzed Diels–Alder reaction (Supplementary Fig. 29).

We attempted to optimize the conditions for the Diels–Alder reaction. First, we varied [Cu^2+^] versus [**BTA**^**BA**^] and found that the *ee* value of the endo isomer reached a maximum at [Cu^2+^]/[**BTA**^**BA**^] = 1.0% (Fig. 3e). When [Cu^2+^]/[**BTA**^**BA**^] exceeded 1.0%, the *ee* value of the endo isomer dropped, most likely because of a possible increase in the amount of Cu^2+^ unanchored to the helical **PBTA**^**BA**^ nanoribbons. A strong base such as NaOH is expected to deprotonate the benzoic acid groups of **PBTA**^**BA**^ to promote the anchoring of Cu^2+^ onto the helical **PBTA**^**BA**^ nanoribbons^36^. In fact, as shown in Fig. 3f, the addition of NaOH to the reaction system resulted in a considerable enhancement of the enantioselectivity of the reaction, where an average *ee* value of >40% was realized at [NaOH]/[**BTA**^**BA**^] = 50% with the highest *ee* value of 46% (major/minor enantiomers = 73/27; Supplementary Fig. 30).

### **C**hirality transfer via electrostatic interaction

**Fig. 4:**
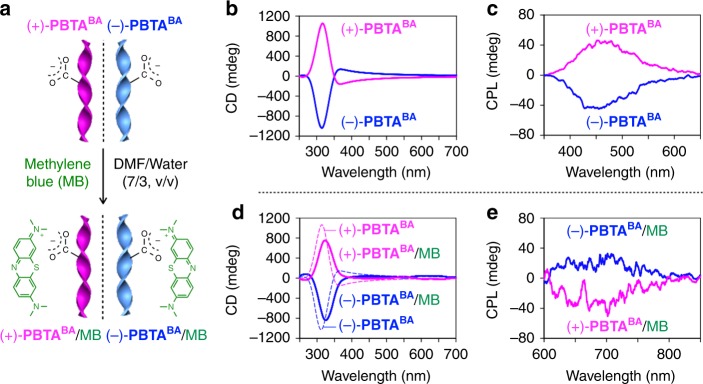
Chiral environment of the cation-binding sites in **PBTA**^**BA**^ nanoribbons. **a** Schematic representation of the binding of methylene blue (MB) onto **PBTA**^**BA**^ at its carboxylate units via an electrostatic interaction. **b**, **c** Circular dichroism (CD, **b**) and circularly polarized luminescence (CPL, *λ*_ext_ = 290 nm, **c**) spectra at 25 °C of DMF/water (7/3, v/v; 1 mL) suspensions of (−)-**PBTA**^**BA**^ (blue) and (+)-**PBTA**^**BA**^ (pink) ([**BTA**^**BA**^] = 5.3 mM, Fig. 2d, e). **d**, **e** CD (**d**) and CPL (*λ*_ext_ = 550 nm, **e**) spectra at 25 °C of DMF/water (7/3, v/v; 1 mL) suspensions of (−)-**PBTA**^**BA**^ (blue) and (+)-**PBTA**^**BA**^ (pink) ([**BTA**^**BA**^] = 5.3 mM) containing MB ([MB]/[**BTA**^**BA**^] = 20%). Source data are provided as a Source Data file

We explored the stereochemical environment of the cation-binding sites in the helical **PBTA**^**BA**^ nanoribbon by using a cationic fluorescent dye, methylene blue (MB, Fig. 4a). When an aqueous solution of MB (21.2 mM, 50 µL) was added to a DMF/water (7/3, v/v; 1 mL) suspension of mirror symmetry-broken (−)-**PBTA**^**BA**^ ([**BTA**^**BA**^] = 5.3 mM, [MB]/[**BTA**^**BA**^] = 20%), a broad positive CD band at 570–700 nm emerged (Fig. 4d and Supplementary Fig. 31). When (+)-**PBTA**^**BA**^ ([**BTA**^**BA**^] = 5.3 mM) was employed instead of (−)-**PBTA** for mixing with MB, the sign of the CD band at 570–700 nm was negative (Fig. 4d and Supplementary Fig. 31). Considering that MB has an absorption band at 570–700 nm (Supplementary Fig. 31), the CD bands in this region are assignable to MB, which is supposedly located in a chiral environment by being bound to the helical nanoribbon of **PBTA**^**BA**^ at its carboxylate units (Fig. 4a)^58,59^. Note that mirror symmetry-broken (−)-**PBTA**^**BA**^/MB and (+)-**PBTA**^**BA**^/MB displayed positive and negative circularly polarized luminescence (CPL) bands at 600–800 nm, respectively (*λ*_ext_ = 550 nm, Fig. 4e and Supplementary Fig. 32b). These CPL bands are obviously different from those observed for (−)-**PBTA**^**BA**^ and (+)-**PBTA**^**BA**^ (400–600 nm, *λ*_ext_ = 290 nm; Fig. 4c and Supplementary Fig. 32a) and therefore are assignable to MB. The luminescence dissymmetry factors (*g*_lum_) of (−)-**PBTA**^**BA**^/MB and (+)-**PBTA**^**BA**^/MB, evaluated from their CPL intensities relative to the fluorescence intensities at 700 nm, were ~10^−2^, which are much larger than those commonly observed for chiral organic compounds (10^−^^5^–10^−^^2^)^60^. This result again indicates unambiguously that MB is localized in a chiral environment provided by (−)-**PBTA**^**BA**^ or (+)-**PBTA**^**BA**^.

## Discussion

All of these results allow us to conclude that a mirror symmetry-broken helical supramolecular nanoribbon composed of an achiral monomer can serve as a chiral ligand for mediating enantioselective catalysis. Although the noncrystalline nature of **PBTA**^**BA**^ (Supplementary Figs. 17 and 18) does not permit elucidation of its molecular packing, the successful CPL observation from MB in Fig. 4e suggests that the catalytic site having a Cu^2+^ ion is chiral. As for the modest enantioselectivity of the reaction, we assume that this is due to the helical nanoribbon structure, which is formed by rolling up of a rather planer, sheet structure. It is likely that the catalytic sites located on such a gently curved surface cannot efficiently bias the stereochemical course of the reaction. We also noted that the mirror symmetry-broken helical nanoribbons of **BTA**^**BA**^ display intense electronic CD (Δ*ε* = ~570 L mol^−1^ cm^−1^). This is very rare, but some helicene nanoribbons are known to show such an amplified chiroptical behavior^61^. The next grand challenge to tackle is how one can obtain a helical catalyst with a preferred handedness by mirror symmetry breaking. Together with our achievement, the design of mirror symmetry breaking may revolutionize the manufacturing process of chiral compounds having many potential applications.

## Methods

### Materials

Unless otherwise noted, reagents and solvents were used as received from commercial sources without further purification. Benzene-1,3,5-tricarbonyl trichloride was purchased from Alfa Aesar. Ethyl 4-aminobenzoate was purchased from Adamas. Copper (II) nitrate (Cu(NO_3_)_2_) was purchased from J&K Scientific. Methylene blue was purchased from Tokyo Chemical Industry (TCI). Dicyclopentadiene was purchased from Acros Organics. Cyclopentadiene was prepared by distillation of commercial dicyclopentadiene at 180 °C. (*E*)-3-Phenyl-1-(pyridin-2-yl)prop-2-en-1-one (aza-chalcone) was prepared according to a previously reported method^56^.

### Characterization

^1^H and ^13^C nuclear magnetic resonance (NMR) spectra were recorded on a JEOL model ALPHA-500 spectrometer in which the chemical shifts for ^1^H NMR spectroscopy were determined with respect to a non-deuterated solvent residue, CHD_2_(CD_3_)SO (*δ* 2.50 ppm), and those for ^13^C NMR spectroscopy were determined with respect to DMSO (*δ* 39.52 ppm). Matrix-assisted laser desorption/ionization time-of-flight mass (MALDI-TOF MS) spectrometry was performed using *α*-cyano-4-hydroxycinnamic acid (CHCA) as a matrix using a Bruker Daltonics Autoflex^TM^ Speed MALDI-TOF/TOF spectrometer. Electronic absorption spectra were recorded in sandwich-type quartz cuvettes with an optical path length of 0.1 mm using a JASCO UV-550 spectrometer. CD and LD spectra were recorded in sandwich-type quartz cuvettes with an optical path length of 0.1 mm using a JASCO J-810 spectrometer. Circularly polarized luminescence (CPL) spectra were recorded in sandwich-type quartz cuvettes with an optical path length of 0.1 mm using a JASCO CPL-200 spectrometer. Fluorescence spectra were recorded using a Hitachi F-4500 spectrophotometer. FT-IR spectra were recorded using a JASCO model FT/IR-6100Plus FT-IR spectrometer. SEM was performed using a Hitachi S-4800 FE-SEM with an accelerating voltage of 10 kV. X-ray diffraction (XRD) analysis was performed on a Rigaku D/Max-2500 X-ray diffractometer (Japan) with Cu Kα radiation (*λ* = 1.5406 Å), which was operated at a voltage of 40 kV and a current of 200 mA. Polarized optical microscopy (POM) was performed using a Leica DM2700 upright materials microscope. Chiral HPLC was performed using a Waters 1525 HPLC system with a Daicel Chiralcel^®^ OD-H column.

## Supplementary information

Supplementary Information

Peer Review

Source Data

## Data Availability

The source data underlying Fig. 3b–f and 4b–e and Supplementary Figs. 1–11, 12a, e, h, 14–16, 18–21, 23, 24b, 25 and 27–32 are provided as a Source Data file, which is available via figshare (DOI: 10.6084/m9.figshare.8940404).
