# Supplementary material for: Asymmetric catalysis mediated by a mirror symmetry-broken helical nanoribbon
Source: Nat Commun. 2019 Sep 4;10:3976. doi: 10.1038/s41467-019-11840-3 (PMC6726595; doi:10.1038/s41467-019-11840-3)
Supplement: Supplementary file 1 — Supplementary Information [file 41467_2019_11840_MOESM1_ESM.pdf]

Supplementary Information

Asymmetric catalysis mediated by a mirror symmetry-broken helical nanoribbon

Zhaocun Shen*, Yutao Sang, Tianyu Wang, Jian Jiang, Yan Meng,

Yuqian Jiang, Kou Okuro*, Takuzo Aida* & Minghua Liu*

Table of Contents

1. Supplementary Methods S2

2. Supplementary Figures S5

3. Supplementary Tables S38

4. Supplementary References S42

1. Supplementary Methods

1.1 Synthesis

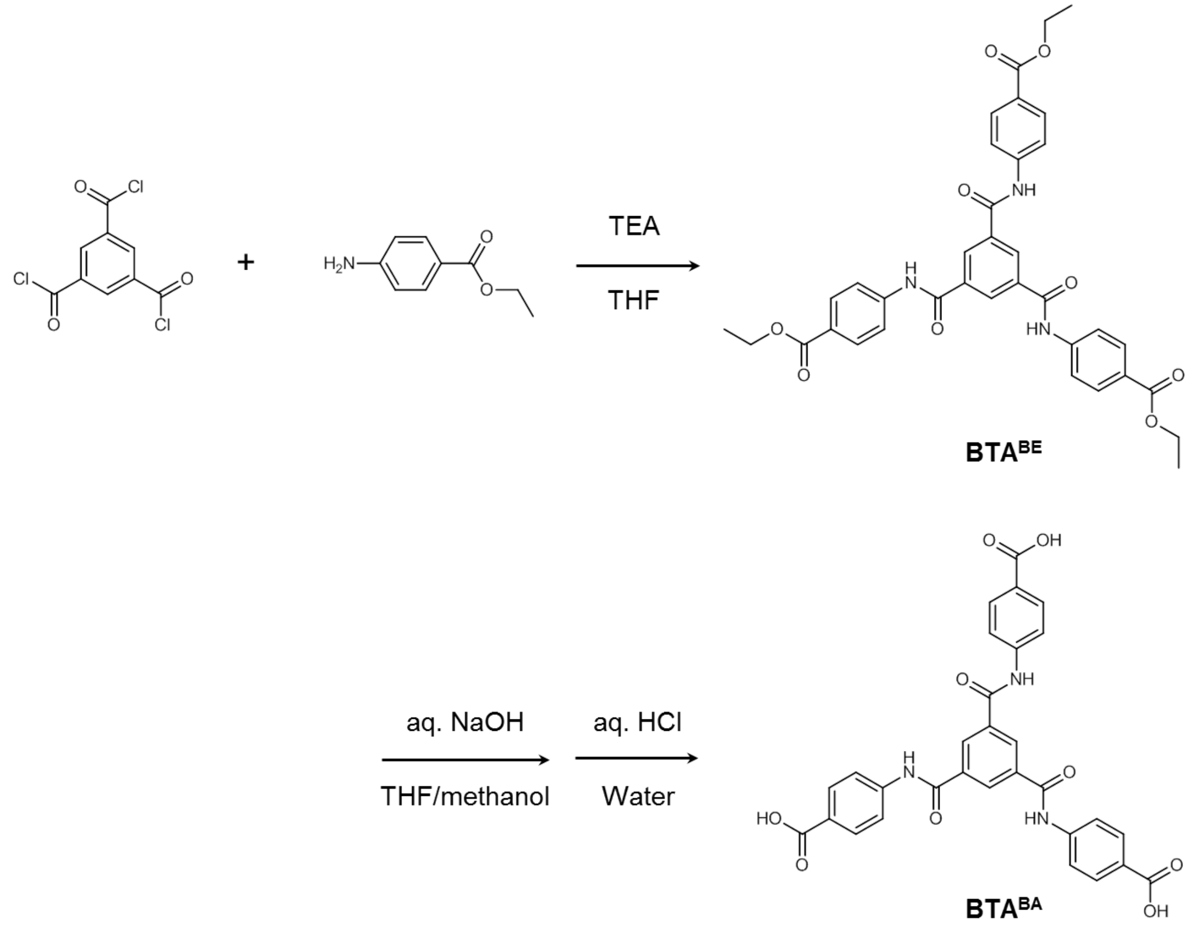

BTA^{BE}. To a THF solution (30 mL) of a mixture of ethyl 4-aminobenzoate (2.64 g, 16.0 mmol) and triethylamine (TEA, 2.2 mL, 16.0 mmol), a THF solution (3 mL) of benzene-1,3,5-tricarbonyl trichloride (1.06 g, 4.0 mmol) was added dropwise, and the mixture was stirred for 12 hours at room temperature. The reaction mixture was filtered off from the insoluble fraction. The filtrate was evaporated to 10 mL under reduced pressure and then slowly added to methanol (100 mL) under stirring. The formed precipitates were filtered, and an insoluble fraction was successively washed with water and methanol and then dried under reduced pressure, affording **BTA^{BE}** as a white solid substance (1.98 g, yield: 76%). ^{1}H NMR (500 MHz, DMSO-d_6; ppm): δ 1.33 (t, J = 7.0 Hz, 9H), 4.31 (q, J = 7.2 Hz, 6H), 8.00 (m, 12H), 8.77 (s, 3H), 10.91 (s, 3H). ^{13}C NMR (125 MHz, DMSO-d_6; ppm): δ 14.3, 60.6, 119.7, 125.0, 130.2, 130.4, 135.2, 143.3, 164.8, 165.4. MALDI-TOF-MS: *m*/*z* found: 674.75 ([M + Na]$^{+}$ calcd: 674.66).

BTA^{BA}. To a THF/methanol (1/1, v/v; 30 mL) suspension of **BTA^{BE}** (1.31 g, 2.0 mmol), an aqueous solution of NaOH (4 M, 7.5 mL) was slowly added, and the mixture was stirred overnight at room temperature. The reaction mixture was diluted with water (60 mL), and an aqueous solution of HCl (1 M) was added to the resultant mixture until pH = 1. The formed precipitates were filtered, and an insoluble fraction was successively washed with water and acetone and then dried under reduced pressure, affording **BTA^{BA}** as a white solid substance (0.70 g, yield: 62%). ^{1}H NMR (500 MHz, DMSO-d_6; ppm): δ 7.98 (m, 12H), 8.75 (s, 3H), 10.89 (s, 3H), 12.81 (broad s, 3H). ^{13}C NMR (125 MHz, DMSO-d_6; ppm): δ 120.0, 126.0, 130.3, 130.4, 135.3, 143.0, 164.9, 167.0. MALDI-TOF-MS: m/z found: 566.36 ([M − H]$^{-}$ calcd: 566.13).

1.2 Preparation of DMF/water Gels of PBTA^{BA}

Typically, **BTA^{BA}** (3 mg, 5.3×10^{-6} mol) was dissolved in *N*,*N*-dimethylformamide (DMF, 0.7 mL) in a 5-mL cylindrical glass vial, and then water (0.3 mL) was added to the solution. The resultant mixture was allowed to stand for 2 hours at 25 °C, affording a DMF/water (7/3, v/v; 1 mL) gel of **PBTA^{BA}** ([**BTA^{BA}**] = 5.3 mM, Fig. 2b). Likewise, DMF/water (7/3, v/v; 1 mL) gels of **PBTA^{BA}** ([**BTA^{BA}**] = 8.8 mM and 17.6 mM), DMF/water (6/4, 5/5, 4/6, 3/7, v/v; 1 mL) gels of **PBTA^{BA}** ([**BTA^{BA}**] = 5.3 mM), and DMF/water (7/3, v/v; 1 mL) gels of **PBTA^{BE}** ([**BTA^{BE}**] = 4.6 mM) were prepared.

1.3 Preparation of CD-Active Suspensions of PBTA^{BA} by Magnetic Rotary Stirring

Typically, a DMF/water (7/3, v/v; 1 mL) gel of **PBTA^{BA}** ([**BTA^{BA}**] = 5.3 mM) prepared according to the procedure in 1.2 was heated at 100 °C for 3 min, affording a clear solution. Then, this solution was allowed to cool to 25 °C with magnetic rotary stirring at 1200 rpm for 40 min using an IKA® RCT basic magnetic stirrer with a 5.0 × 10.0 mm Teflon-coated stirring bar, affording a white suspension containing mirror symmetry-broken helical **PBTA^{BA}** nanoribbons. Here, the direction of rotary stirring did not determine the dominant handedness of **PBTA^{BA}** but either (*P*)-dominant **PBTA^{BA}** (Fig. 2d) or (*M*)-dominant **PBTA^{BA}** (Fig. 2e) appeared stochastically.

1.4 Diels–Alder Reaction with PBTA^{BA}

Typically, to a DMF/water (7/3, v/v; 1 mL) suspension of (–)-**PBTA^{BA}** or (+)-**PBTA^{BA}** ([**BTA^{BA}**] = 5.3 mM) prepared according to the procedure in 1.3, an aqueous solution of $Cu(NO_3)_2$ was added (0.82, 4.1, 20.5 and 41 mM, 13 μL; [$Cu(NO_3)_2$]/[**BTA^{BA}**] = 0.2%, 1%, 5% and 10%, respectively) under stirring, and the mixture was stirred for 1 hour at 25 °C. Then, an acetonitrile (40 μL) solution of aza-chalcone (1 mg) and freshly distilled cyclopentadiene (40 μL) were successively added to the mixture under stirring, and the resultant mixture was stirred at 1200 rpm using an IKA® RCT basic magnetic stirrer with a 5.0 × 10.0 mm Teflon-coated stirring bar at 25 °C for 36 hours. Then, water (2 mL) was added to the reaction mixture, and the mixture was extracted with ethyl acetate (0.5 mL). The organic extract, after being evaporated to dryness, was subjected to chiral HPLC to evaluate the enantiomeric excess (*ee*) values of the products[1].

1.5 Calculation of the Dissymmetry Factors g_{abs} and g_{lum}

The absorption dissymmetry factor (g_{abs}) is defined as $g_{abs} = (\varepsilon_L - \varepsilon_R)/(\varepsilon_L + \varepsilon_R)$, where ε_L and ε_R refer to the extinction coefficients for the left- and right-handed circularly polarized light, respectively. Experimentally, CD was measured using a JASCO J-810 spectrometer, and the value of g_{abs} was calculated using the equation g_{abs} = [ellipticity/32980]/Abs, where the value of 32980 is the ellipticity to the absorbance conversion factor and Abs is the absorbance at the CD extremum.

The luminescence dissymmetry factor (g_{lum}) is defined as $g_{lum} = 2 \times (I_L - I_R)/(I_L + I_R)$, where I_L and I_R refer to the intensities of the left- and right-handed CPL, respectively. The maximum g_{lum} value ranges from +2 for an ideal left CPL to −2 for an ideal right CPL, whereas $g_{lum} = 0$ corresponds to no circular polarization of the luminescence. Experimentally, CPL was measured using a JASCO CPL-200 spectrometer, and the value of g_{lum} was calculated using the equation g_{lum} = 2 × [ellipticity/(32980/ln10)]/FL, where FL is the fluorescence intensity at the CPL extremum.

2. Supplementary Figures

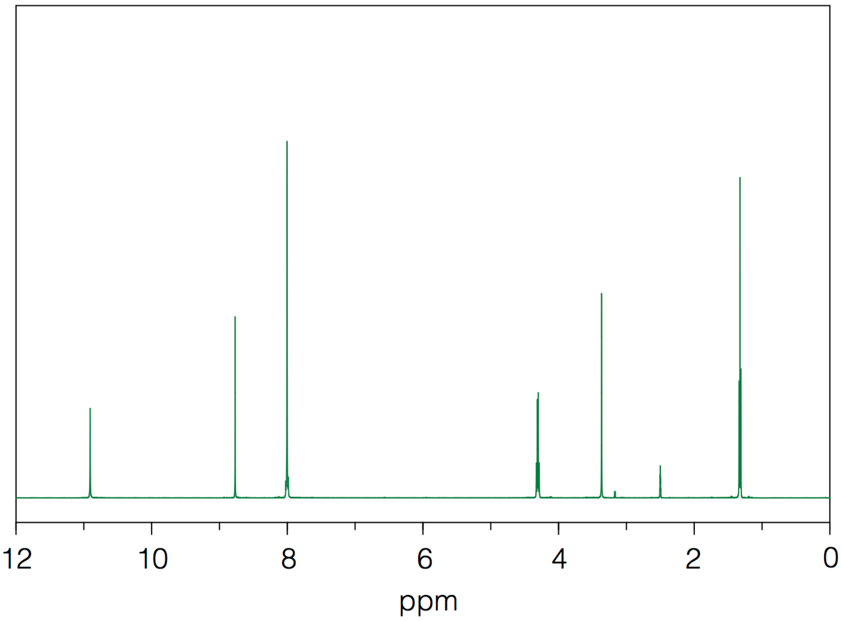

Supplementary Fig. 1 | ^{1}H NMR spectroscopy of BTA^{BE}. ^{1}H NMR spectrum (500 MHz) of **BTA^{BE}** in DMSO-d_6 at 25 °C. The signals at δ 2.50 and 3.33 ppm are due to DMSO and water, respectively. Source data are provided as a Source Data file.

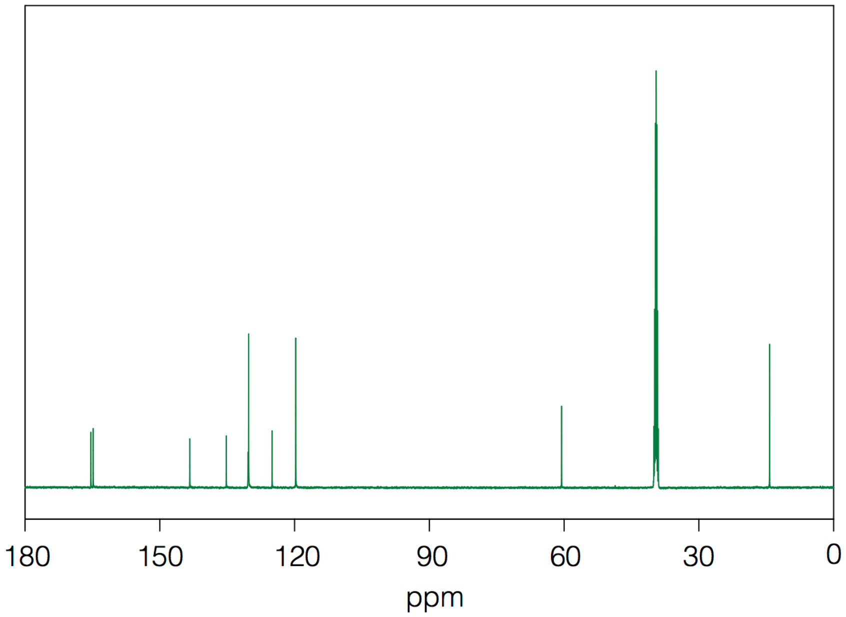

Supplementary Fig. 2 | ^{13}C NMR spectroscopy of BTA^{BE}. ^{13}C NMR spectrum (125 MHz) of **BTA^{BE}** in DMSO-d_6 at 25 °C. The signal at δ 39.52 ppm is due to DMSO. Source data are provided as a Source Data file.

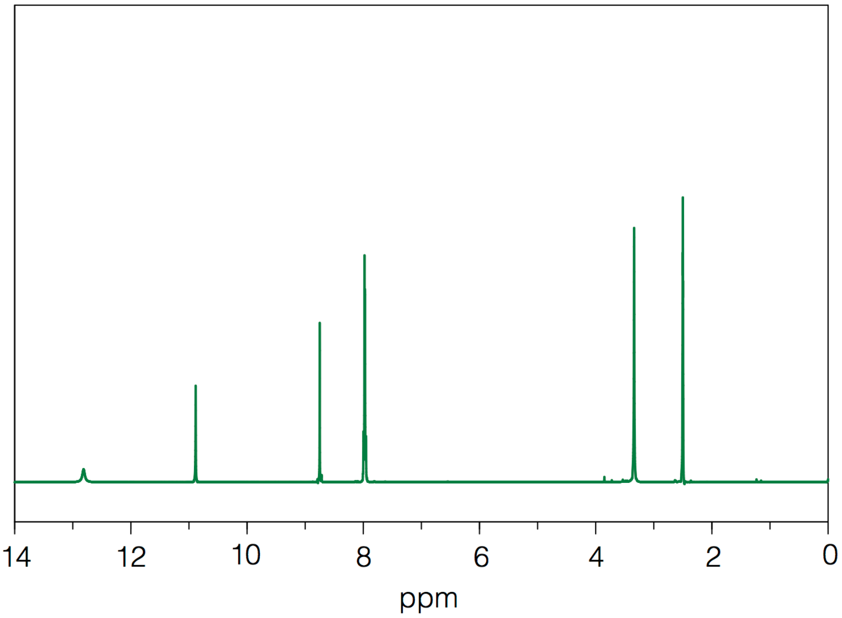

Supplementary Fig. 3 | ^{1}H NMR spectroscopy of BTA^{BA}. ^{1}H NMR spectrum (500 MHz) of **BTA^{BA}** in DMSO-d_6 at 25 °C. The signals at δ 2.50 and 3.33 ppm are due to DMSO and water, respectively. Source data are provided as a Source Data file.

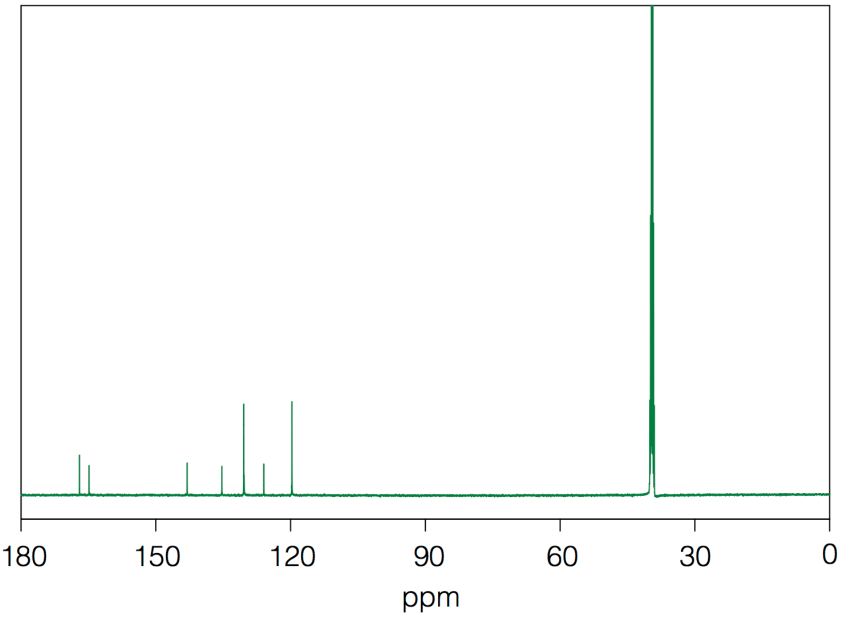

Supplementary Fig. 4 | ^{13}C NMR spectroscopy of BTA^{BA}. ^{13}C NMR spectrum (125 MHz) of **BTA^{BA}** in DMSO-d_6 at 25 °C. The signal at δ 39.52 ppm is due to DMSO. Source data are provided as a Source Data file.

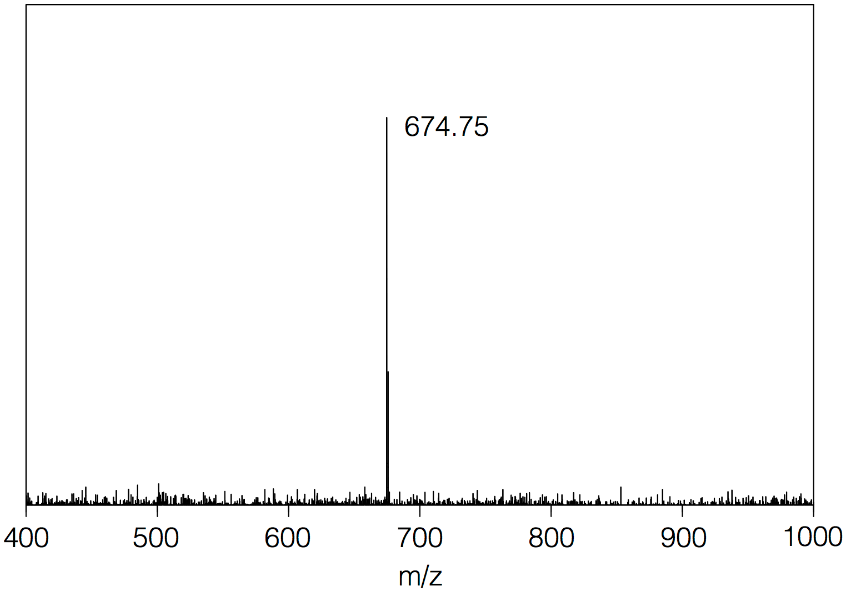

Supplementary Fig. 5 | MALDI-TOF mass spectrometry of BTA^{BE}. MALDI-TOF mass spectrum of **BTA^{BE}** using CHCA as the matrix. Source data are provided as a Source Data file.

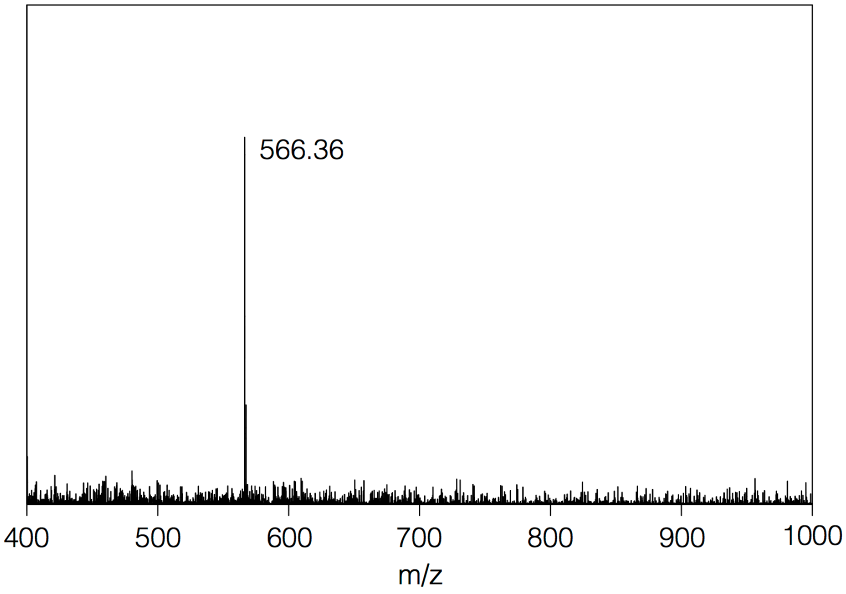

Supplementary Fig. 6 | MALDI-TOF mass spectrometry of BTA^{BA}. MALDI-TOF mass spectrum of **BTA^{BA}** using CHCA as the matrix. Source data are provided as a Source Data file.

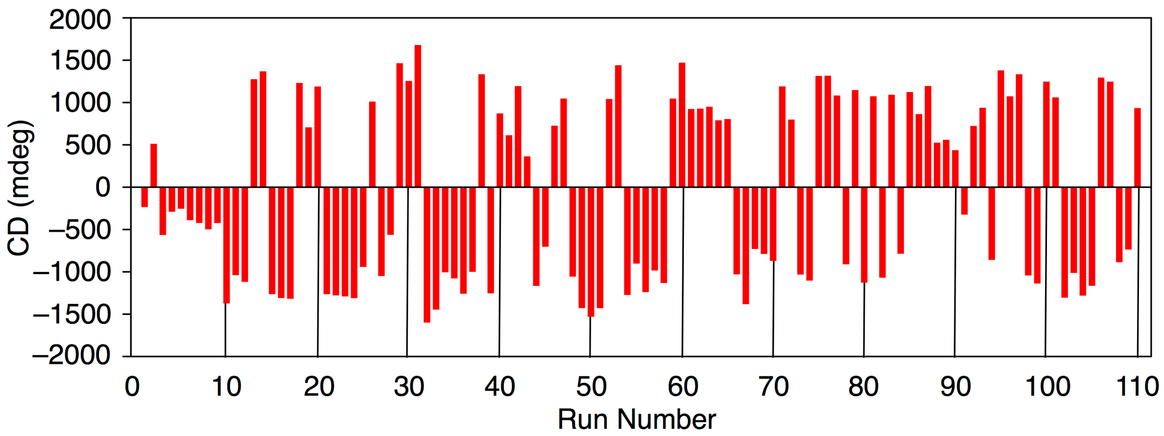

Supplementary Fig. 7 | Rotary stirring effects on mirror symmetry breaking of PBTA^{BA}. Statistical CD intensities at 316 nm obtained from a total 110 samples of DMF/water (7/3, v/v; 1 mL) suspensions of **PBTA^{BA}** ([**BTA^{BA}**] = 5.3 mM) prepared according to the procedure in 1.3 with clockwise magnetic rotary stirring. For all the 110 samples, the absorbance at 290 nm was normalized as 1.0. Source data are provided as a Source Data file.

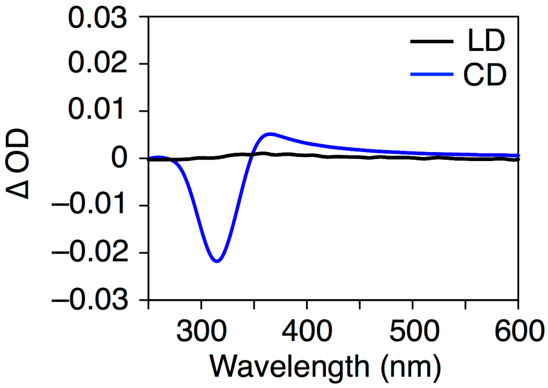

Supplementary Fig. 8 | LD artifacts on CD of the suspension of PBTA^{BA}. CD (blue) and LD (black) spectra at 25 °C of a DMF/water (7/3, v/v; 1 mL) suspension of (–)-**PBTA**^{BA} ([**BTA**^{BA}] = 5.3 mM). Source data are provided as a Source Data file.

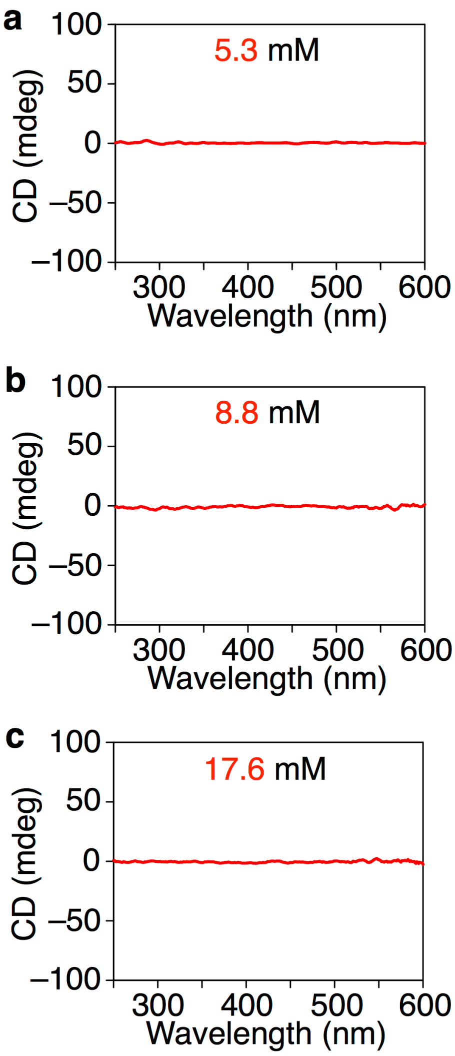

Supplementary Fig. 9 | CD spectroscopy of gels at different concentrations. **a**–**c**, CD spectra at 25 °C of DMF/water (7/3, v/v; 1 mL) gels of **PBTA**$^{\mathbf{BA}}$ at [**BTA**$^{\mathbf{BA}}$] = 5.3 mM (3 mg/mL, **a**), 8.8 mM (5 mg/mL, **b**), and 17.6 mM (10 mg/mL, **c**), respectively. Source data are provided as a Source Data file.

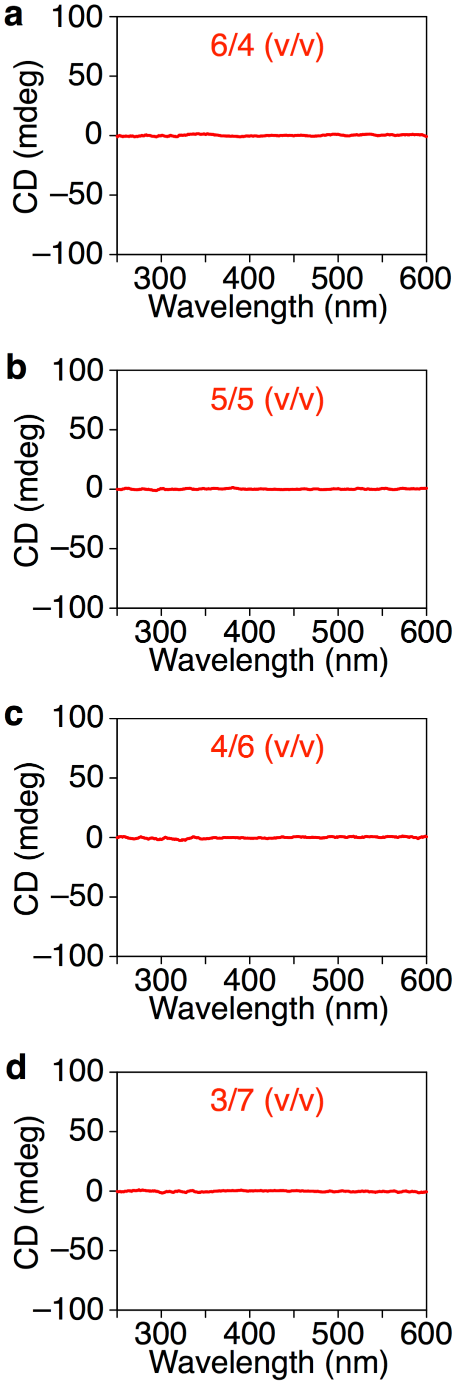

Supplementary Fig. 10 | CD spectroscopy of gels at different solvent volume ratios. a–d, CD spectra at 25 °C of DMF/water (1 mL) gels of **PBTA**$^{\mathbf{BA}}$ ([**BTA**$^{\mathbf{BA}}$] = 5.3 mM) at different solvent volume ratios (v/v): 6/4 (**a**), 5/5 (**b**), 4/6 (**c**) and 3/7 (**d**). Source data are provided as a Source Data file.

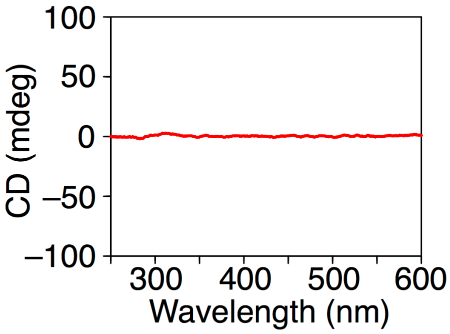

Supplementary Fig. 11 | CD spectroscopy of a precipitate obtained without rotary stirring. CD spectra at 25 °C of a precipitate of **PBTA^{BA}** ([**BTA^{BA}**] = 5.3 mM, Fig. 2f) in DMF/water (7/3, v/v; 1 mL) prepared by natural cooling of a clear, hot DMF/water (7/3, v/v; 1 mL) solution of **BTA^{BA}** (5.3 mM, Fig. 2c) without magnetic rotary stirring. Source data are provided as a Source Data file.

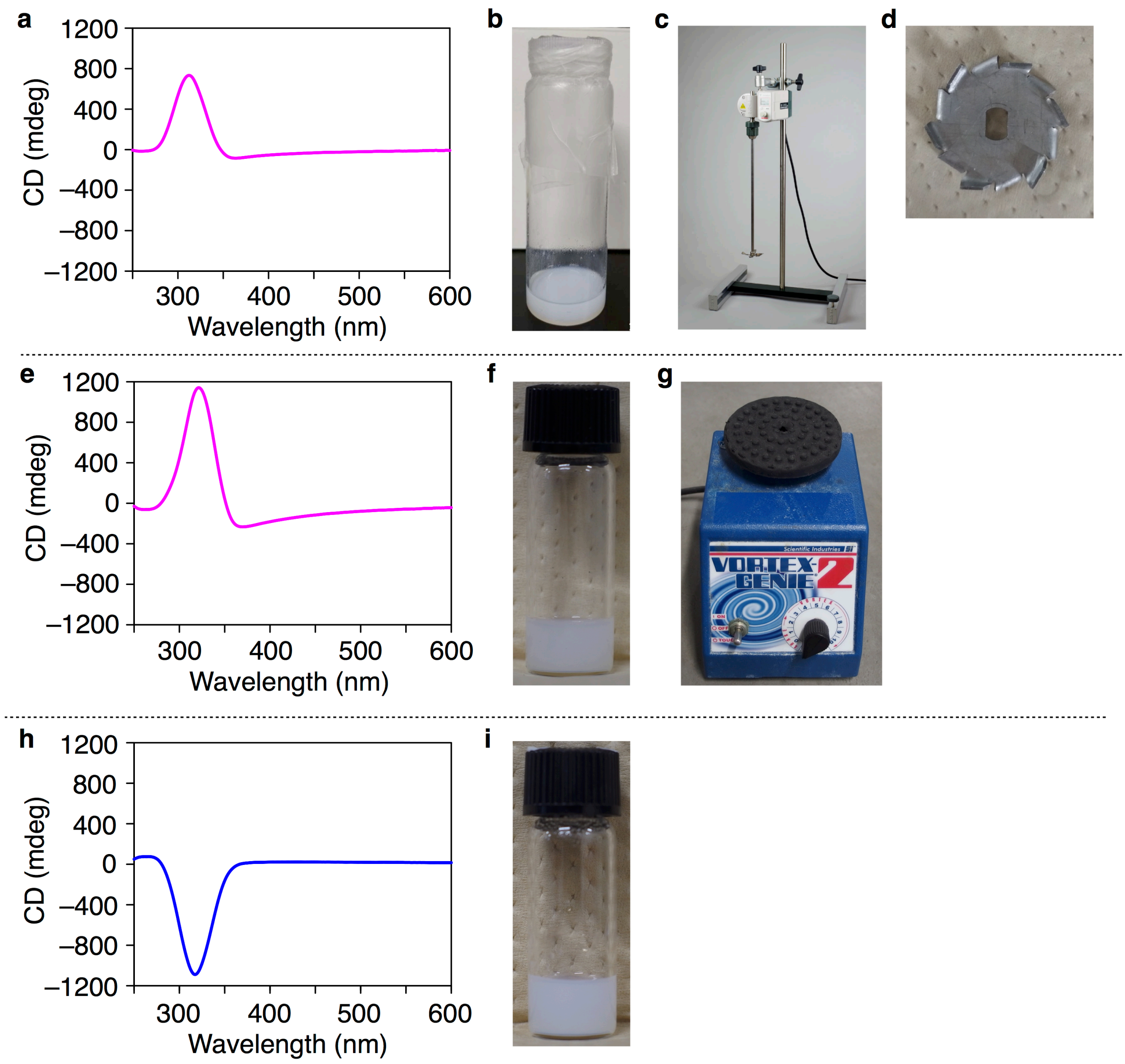

Supplementary Fig. 12 | Mirror symmetry breaking induced by other agitation methods. **a**–**d**, CD spectrum (**a**) at 25 °C of a DMF/water (7/3, v/v; 10 mL) suspension (**b**) of **PBTA^{BA}** ([**BTA^{BA}**] = 5.3 mM) prepared using a 100-mL cylindrical glass vial by mechanical rotary stirring at 1200 rpm for 40 min using a HEIDON BL1200 mechanical stirrer (**c**) with a stirring impellor (diameter: 25 mm; **d**) under otherwise identical conditions to the procedure in 1.3. **e**–**g**, CD spectrum (**e**) at 25 °C of a DMF/water (7/3, v/v; 1 mL) suspension (**f**) of **PBTA^{BA}** ([**BTA^{BA}**] = 5.3 mM) prepared by vortex mixing for 40 min using a Scientific Industries Vortex-Genie 2 vortex mixer (**g**) under otherwise identical conditions to the procedure in 1.3. **h**, **i**, CD spectrum (**h**) at 25 °C of a DMF/water (7/3, v/v; 1 mL) suspension (**i**) of **PBTA^{BA}** ([**BTA^{BA}**] = 5.3 mM) prepared by sonication for 40 min under otherwise identical conditions to the procedure in 1.3. Source data are provided as a Source Data file.

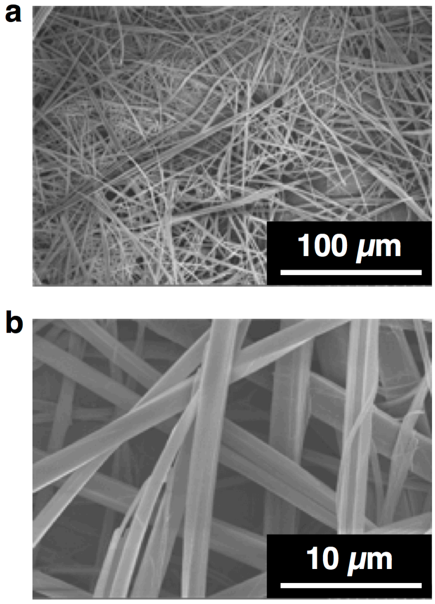

Supplementary Fig. 13 | SEM characterization of the non-twisted PBTA^{BE} fibers. SEM images of an air-dried specimen of a DMF/water (7/3, v/v; 1 mL) gel of **PBTA^{BE}** ([**BTA^{BE}**] = 4.6 mM).

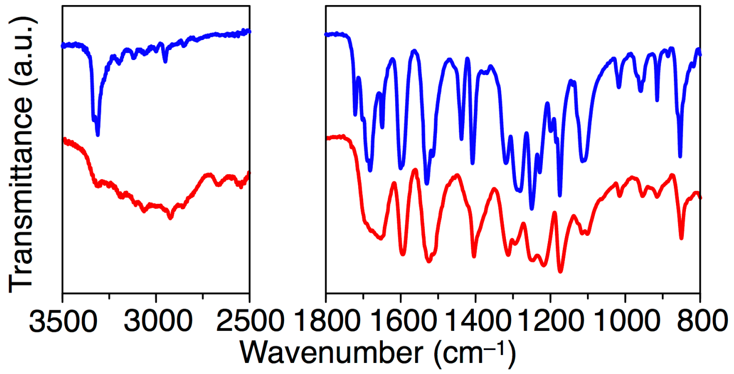

Supplementary Fig. 14 | FT-IR characterization of PBTA^{BA} and PBTA^{BE}. FT-IR spectra at 25 °C of xerogels of **PBTA^{BA}** (red) and **PBTA^{BE}** (blue), prepared by drying DMF/water (7/3, v/v; 1 mL) gels of **PBTA^{BA}** ([**BTA^{BA}**] = 5.3 mM) and **PBTA^{BE}** ([**BTA^{BE}**] = 4.6 mM), respectively, under reduced pressure. Source data are provided as a Source Data file.

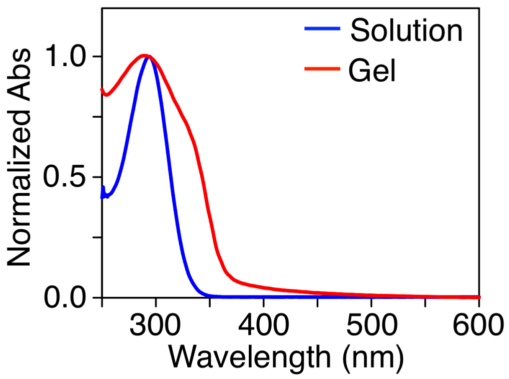

Supplementary Fig. 15 | Absorption spectral characterization of the BTA^{BA} monomer and PBTA^{BA}. Normalized electronic absorption spectra at 25 °C of a DMF solution of **BTA^{BA}** (5.3 mM, blue) and a DMF/water (7/3, v/v; 1 mL) gel of **PBTA^{BA}** ([**BTA^{BA}**] = 5.3 mM, red). Source data are provided as a Source Data file.

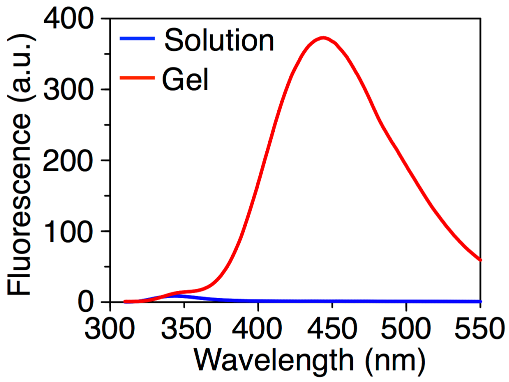

Supplementary Fig. 16 | Fluorescence spectral characterization of the BTA^{BA} monomer and PBTA^{BA}. Fluorescence emission spectra (λ_{ext} = 290 nm) at 25 °C of a DMF (1 mL) solution of **BTA^{BA}** (5.3 mM, blue) and a DMF/water (7/3, v/v; 1 mL) gel of **PBTA^{BA}** ([**BTA^{BA}**] = 5.3 mM, red). Source data are provided as a Source Data file.

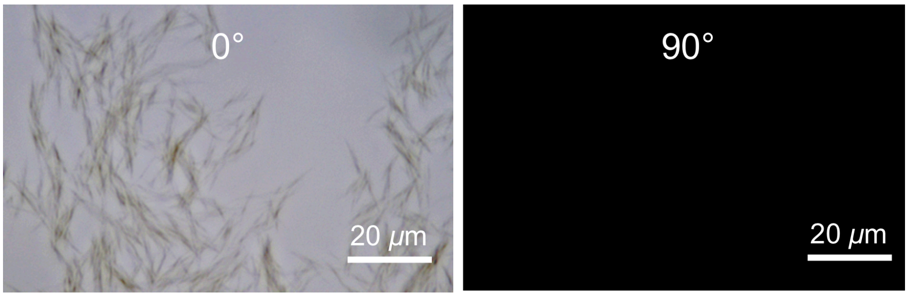

Supplementary Fig. 17 | Polarized optical microscopy (POM) images of the suspension. POM images at 25 °C of a DMF/water (7/3, v/v; 1 mL) suspension of $\mathbf{PBTA^{BA}}$ under parallel and crossed polarizers.

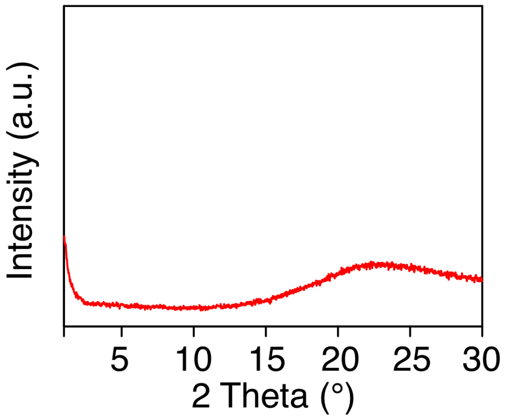

Supplementary Fig. 18 | XRD characterization of PBTA^{BA}. XRD profile at 25 °C of a DMF/water (7/3, v/v; 1 mL) suspension of **PBTA^{BA}** after centrifugation. Source data are provided as a Source Data file.

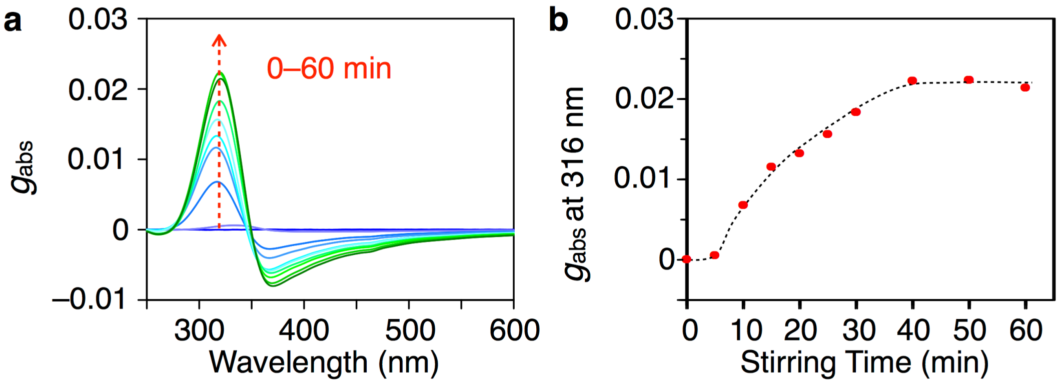

Supplementary Fig. 19 | Effects of stirring time on mirror symmetry breaking of PBTA^{BA}. Absorption dissymmetry factors (g_{abs}) at 250–600 nm (**a**) and g_{abs} at 316 nm (**b**) of the system from 0 to 60 min upon naturally cooling a clear, hot DMF/water (7/3, v/v; 1 mL) solution of **BTA^{BA}** (5.3 mM, Fig. 2c) with magnetic rotary stirring at 1200 rpm. Source data are provided as a Source Data file.

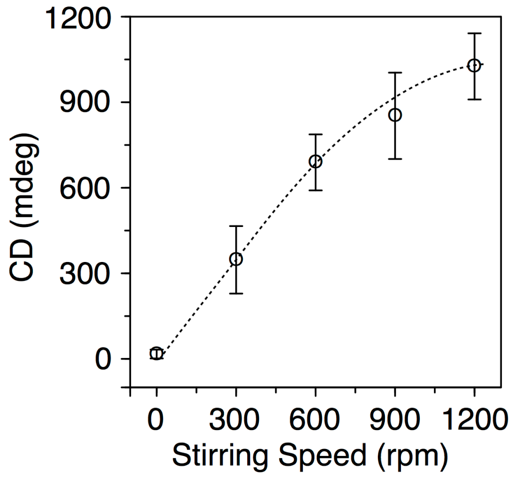

Supplementary Fig. 20 | Effects of stirring speed on mirror symmetry breaking of PBTA^{BA}. CD intensities at 316 nm of DMF/water (7/3, v/v; 1 mL) suspensions of **PBTA^{BA}** ([**BTA^{BA}**] = 5.3 mM) prepared according to the procedure in 1.3 with stirring speed at 0–1200 rpm. Error bars represent the standard deviation. Source data are provided as a Source Data file.

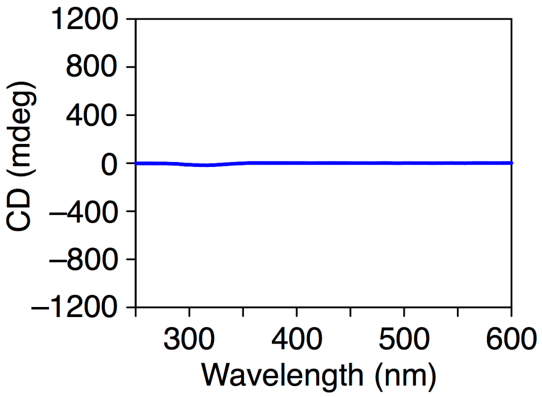

Supplementary Fig. 21 | CD spectroscopy of a suspension obtained by rotary stirring of a gel of PBTA^{BA}. CD spectrum at 25 °C of a DMF/water (7/3, v/v; 1 mL) suspension of **PBTA^{BA}** ([**BTA^{BA}**] = 5.3 mM) prepared by magnetic rotary stirring of a DMF/water (7/3, v/v; 1 mL) gel of **PBTA^{BA}** ([**BTA^{BA}**] = 5.3 mM) at 1200 rpm for 40 min. Source data are provided as a Source Data file.

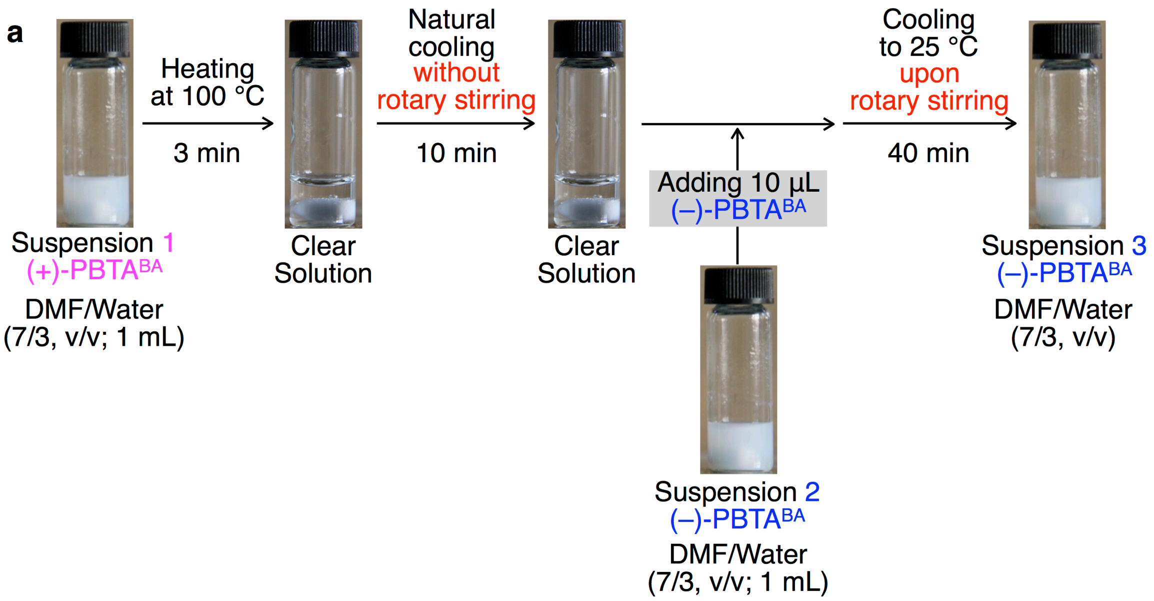

b

Entry	CD of Suspension 1	CD of Suspension 2	CD of Suspension 3
1	+1573	–1104	–1045
2	+942	–1104	–1062
3	+1525	–1104	–904
4	+1232	–1104	–810
5	+1114	–1104	–873
6	+570	–1104	–668
7	+818	–1104	–1593
8	+758	–1104	–969
9	+973	–1104	–1008
10	+1105	–1104	–1283

Supplementary Fig. 22 | Seeded growth of the helical PBTA^{BA} nanoribbons. **a**, Experimental procedure for the preparation of DMF/water (7/3, v/v) suspensions of **PBTA^{BA}** ([**BTA^{BA}**] = 5.3 mM) with predetermined CD signs by the addition of a DMF/water (7/3, v/v; 10 μL) suspension of (–)-**PBTA^{BA}** ([**BTA^{BA}**] = 5.3 mM) into a DMF/water (7/3, v/v; 1 mL) solution of **BTA^{BA}** (5.3 mM) before magnetic rotary stirring. Stirring speed: 1200 rpm. **b**, CD intensities (mdeg) at 316 nm of the used and resultant DMF/water (7/3, v/v) suspensions of **PBTA^{BA}** ([**BTA^{BA}**] = 5.3 mM) in ten parallel experiments.

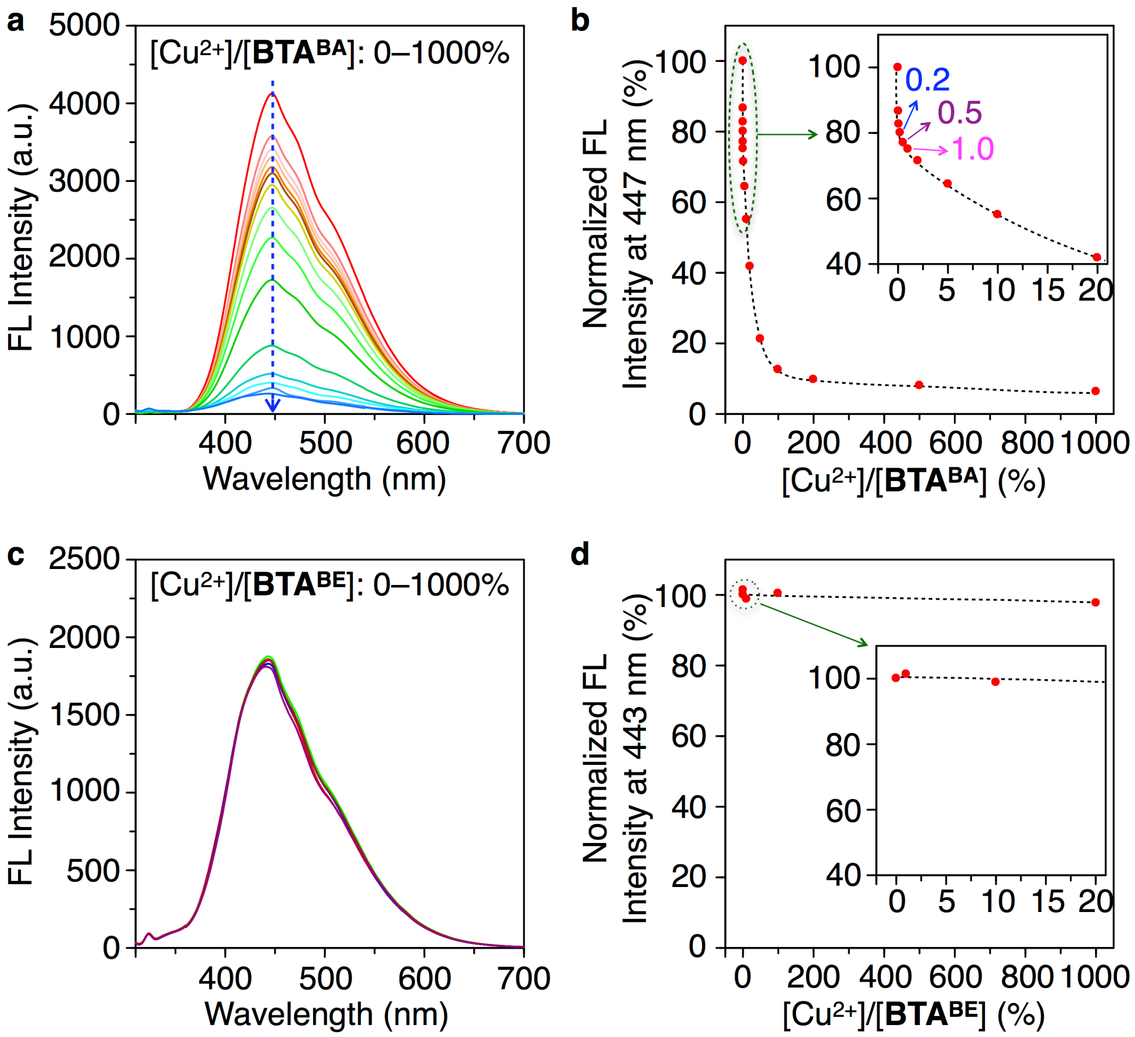

Supplementary Fig. 23 | Cu^{2+} coordination to PBTA^{BA} as studied by fluorescence titration. **a**, **b**, Fluorescence spectral changes (λ_{ext} = 290 nm, **a**) at 25 °C upon titration of a CD-active DMF/water (2 mL) suspension of **PBTA^{BA}** ([**BTA^{BA}**] = 0.053 mM) with an aqueous solution of $Cu(NO_3)_2$ at [Cu^{2+}]/[**BTA^{BA}**] = 0–1000%, and the normalized fluorescence emission intensity at 447 nm (**b**) as a function of [Cu^{2+}]/[**BTA^{BA}**]. **c**, **d**, Fluorescence spectral changes (λ_{ext} = 290 nm, **c**) at 25 °C upon titration of a CD-silent DMF/water (2 mL) suspension of **PBTA^{BE}** ([**BTA^{BE}**] = 0.053 mM) with an aqueous solution of $Cu(NO_3)_2$ at [Cu^{2+}]/[**BTA^{BE}**] = 0–1000%, and the normalized fluorescence emission intensity at 443 nm (**d**) as a function of [Cu^{2+}]/[**BTA^{BE}**]. Source data are provided as a Source Data file.

=> Since **BTA^{BA}** is insoluble in water, a DMF/water (7/3, v/v; 20 μL) suspension of **PBTA^{BA}** ([**BTA^{BA}**] = 5.3 mM) was diluted 100 times with water (1980 μL) to obtain a DMF/water (2 mL) suspension of **PBTA^{BA}** ([**BTA^{BA}**] = 0.053 mM) that remains CD-active and is

suitable for the fluorescence titration experiments. The **PBTA^{BE}** sample was likewise prepared.

=> As shown in Supplementary Fig. 21, the luminescence of **PBTA^{BA}** was quenched upon being mixed with $Cu(NO_3)_2$, whereas the luminescence of **PBTA^{BE}** was quenched negligibly. These results suggest that **PBTA^{BA}** accommodates Cu^{2+} presumably at the peripheral carboxylate units rather than the amide groups.

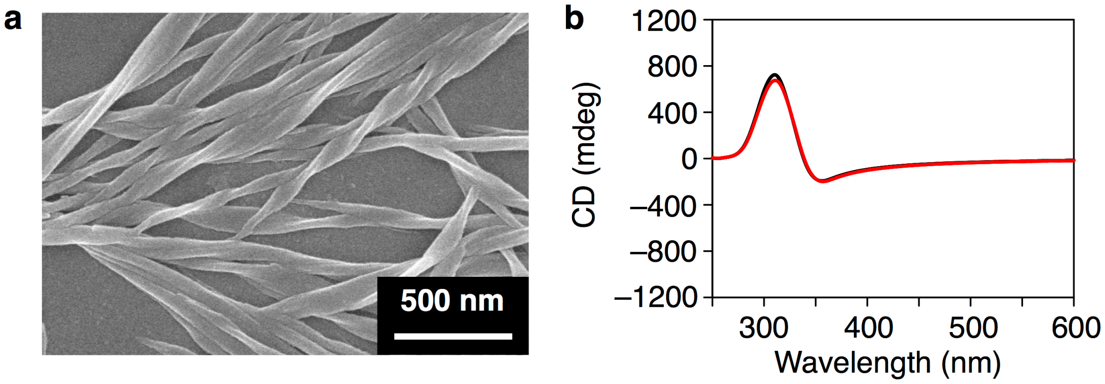

Supplementary Fig. 24 | SEM and CD characterizations of the helical PBTA^{BA}/Cu^{2+} nanoribbons. **a**, SEM image of an air-dried specimen of a DMF/water (7/3, v/v; 1 mL) suspension of (+)-**PBTA**^{BA}/Cu^{2+} ([**BTA**^{BA}] = 5.3 mM, [Cu^{2+}]/[**BTA**^{BA}] = 1.0%). **b**, CD spectra at 25 °C of a DMF/water (7/3, v/v; 1 mL) suspension of (+)-**PBTA**^{BA} ([**BTA**^{BA}] = 5.3 mM) before (black) and after (red) the addition of an aqueous solution of $Cu(NO_3)_2$ ([Cu^{2+}]/[**BTA**^{BA}] = 1.0%). Source data are provided as a Source Data file.

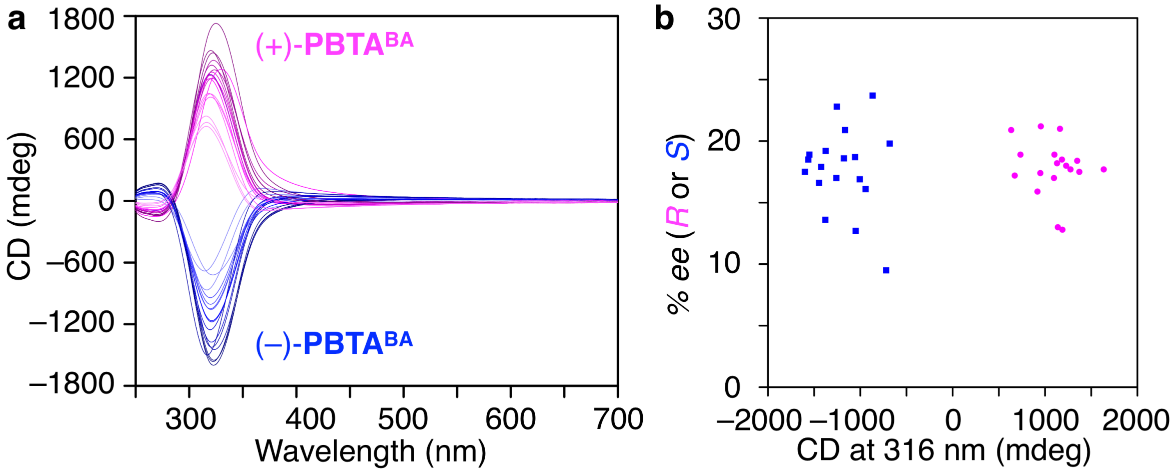

Supplementary Fig. 25 | Enantioselectivity of mirror-symmetry broken PBTA^{BA} in the Cu^{2+}-mediated Diels–Alder reaction. **a**, CD spectra of 36 different batches of DMF/water (7/3, v/v; 1 mL) suspensions of (–)-**PBTA^{BA}** or (+)-**PBTA^{BA}**. **b**, Correlation between CD intensities at 316 nm of the DMF/water (7/3, v/v; 1 mL) suspensions of (–)-**PBTA^{BA}** or (+)-**PBTA^{BA}** and the *ee* values of the endo isomers obtained from the Diels–Alder reaction catalyzed at 25 °C by (–)-**PBTA^{BA}**/Cu^{2+} or (+)-**PBTA^{BA}**/Cu^{2+} (36 separately prepared samples) at [Cu^{2+}]/[**BTA^{BA}**] = 1.0% in DMF/water (7/3, v/v; 1 mL). Source data are provided as a Source Data file.

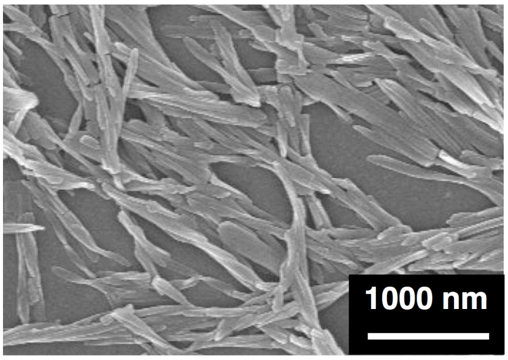

Supplementary Fig. 26 | SEM of the helical PBTA^{BA}/Cu^{2+} nanoribbons after the catalytic reaction. SEM image of an air-dried specimen of a DMF/water (7/3, v/v; 1 mL) suspension of (+)-**PBTA^{BA}**/Cu^{2+} ([**BTA^{BA}**] = 5.3 mM, [Cu^{2+}]/[**BTA^{BA}**] = 1.0%) after the catalytic reaction.

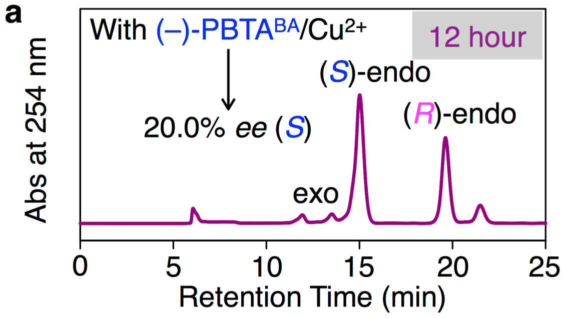

Product	RT (min)	Area	%Area
(*S*)-endo	15.01	149583	60.00
(*R*)-endo	19.62	99875	40.00

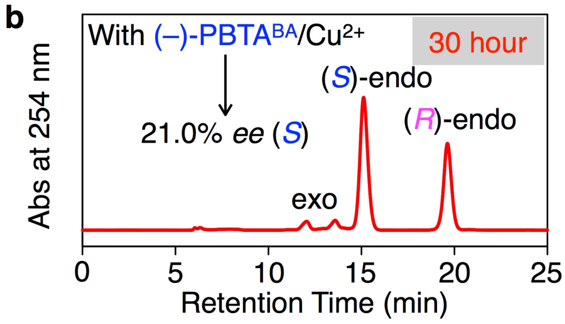

Product	RT (min)	Area	%Area
(*S*)-endo	15.12	246011	60.50
(*R*)-endo	19.63	160543	39.50

Supplementary Fig. 27 | Enantioselectivities of the Diels–Alder reaction in 12 and 30 hours. **a**, **b**, Chiral HPLC traces of the Diels–Alder reaction mixtures catalyzed at 25 °C by (–)-**PBTA**$^{\mathbf{BA}}$/Cu^{2+} at [Cu^{2+}]/[**BTA**$^{\mathbf{BA}}$] = 1.0% in 12 (**a**) and 30 (**b**) hours in DMF/water (7/3, v/v; 1 mL). Source data are provided as a Source Data file.

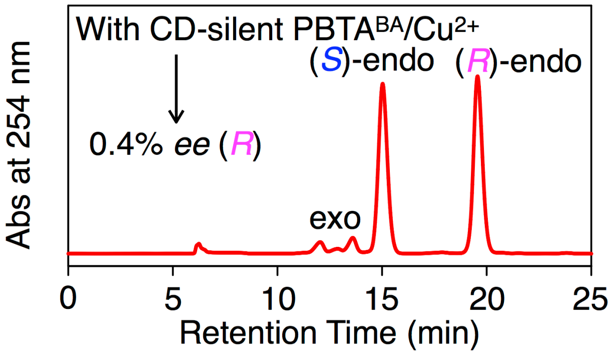

Product	RT (min)	Area	%Area
(*S*)-endo	15.14	2567007	49.80
(*R*)-endo	19.63	2589670	50.20

Supplementary Fig. 28 | Enantioselectivity of the Diels–Alder reaction catalyzed by CD-silent PBTA^{BA}/Cu^{2+}. Chiral HPLC trace of a Diels–Alder reaction mixture catalyzed at 25 °C by virtually CD-silent **PBTA^{BA}**/Cu^{2+} nanoribbons at [Cu^{2+}]/[**BTA^{BA}**] = 1.0% in DMF/water (7/3, v/v; 1 mL). CD-silent **PBTA^{BA}**/Cu^{2+} nanoribbons as the catalyst was prepared by mixing an aqueous solution of $Cu(NO_3)_2$ (4.1 mM, 13 μL) and a CD-silent DMF/water (7/3, v/v; 1 mL) suspension of **PBTA^{BA}** ([**BTA^{BA}**] = 5.3 mM, Supplementary Fig. 19). Source data are provided as a Source Data file.

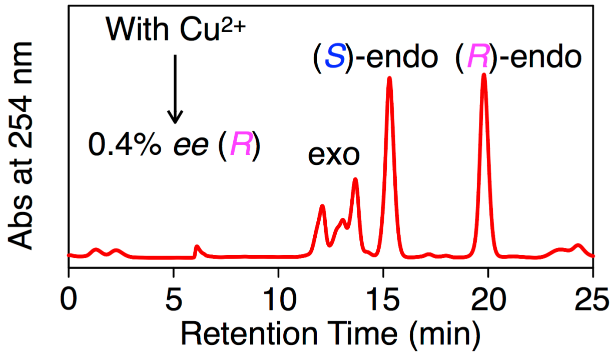

Product	RT (min)	Area	%Area
(*S*)-endo	15.30	4290071	49.80
(*R*)-endo	19.80	4327415	50.20

Supplementary Fig. 29 | Enantioselectivity of the Diels–Alder reaction catalyzed by Cu^{2+}. Chiral HPLC trace of a Diels–Alder reaction mixture catalyzed at 25 °C by $Cu(NO_3)_2$ (0.053 mM) in water (1 mL). Source data are provided as a Source Data file.

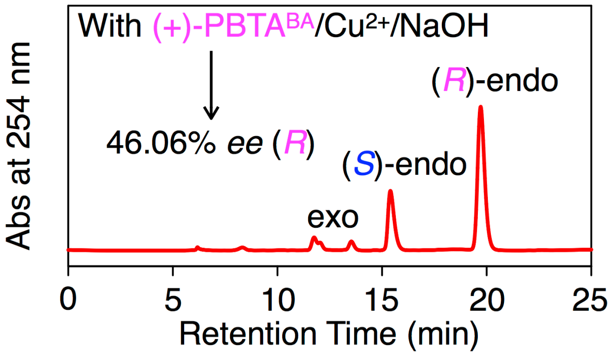

Product	RT (min)	Area	%Area
(*S*)-endo	15.40	1545556	26.97
(*R*)-endo	19.71	4185868	73.03

Supplementary Fig. 30 | The best optimized Diels–Alder reaction catalyzed by PBTA^{BA}/Cu^{2+}. Chiral HPLC trace of a Diels–Alder reaction mixture catalyzed at 25 °C by (+)-**PBTA^{BA}**/Cu^{2+} at [Cu^{2+}]/[**BTA^{BA}**] = 1.0% and [NaOH]/[**BTA^{BA}**] = 50.0% in DMF/water (7/3, v/v; 1 mL), where NaOH was added to the suspension of (+)-**PBTA^{BA}** prior to the addition of $Cu(NO_3)_2$. Source data are provided as a Source Data file.

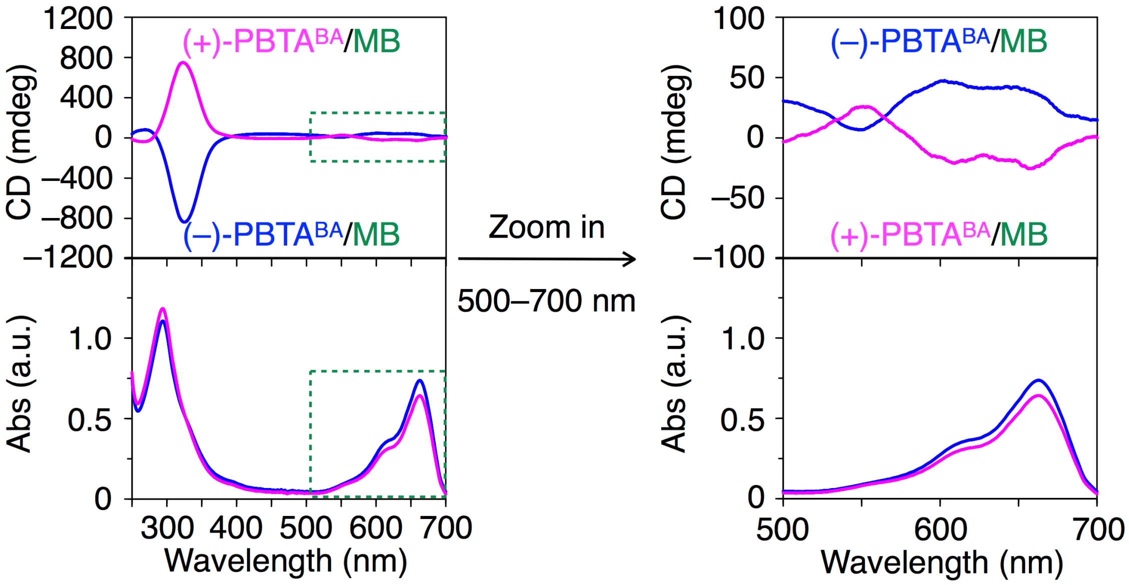

Supplementary Fig. 31 | CD spectroscopy of PBTA^{BA}/methylene blue (MB). CD (upper) and electronic absorption (lower) spectra at 25 °C of DMF/water (7/3, v/v; 1 mL) suspensions of (–)-**PBTA^{BA}** (blue) and (+)-**PBTA^{BA}** (pink) ([**BTA^{BA}**] = 5.3 mM) containing MB ([MB]/[**BTA^{BA}**] = 20%). Source data are provided as a Source Data file.

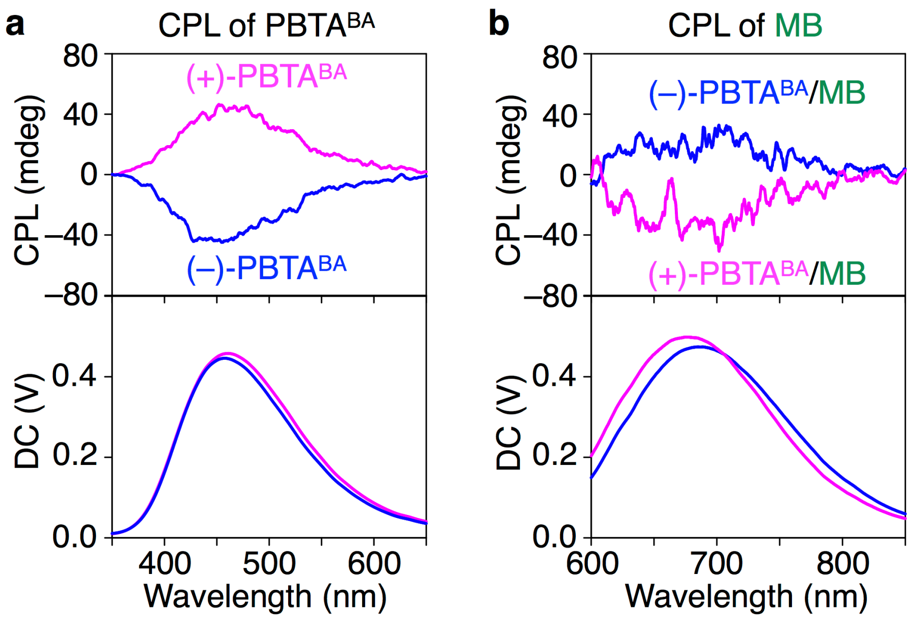

Supplementary Fig. 32 | CPL spectroscopy of PBTA^{BA} and PBTA^{BA}/MB. **a**, CPL spectra (λ_{ext} = 290 nm) at 25 °C of DMF/water (7/3, v/v; 1 mL) suspensions of (–)-**PBTA^{BA}** (blue) and (+)-**PBTA^{BA}** (pink) ([**BTA^{BA}**] = 5.3 mM). **b**, CPL spectra (λ_{ext} = 550 nm) at 25 °C of DMF/water (7/3, v/v; 1 mL) suspensions of (–)-**PBTA^{BA}** (blue) and (+)-**PBTA^{BA}** (pink) ([**BTA^{BA}**] = 5.3 mM) containing MB ([MB]/[**BTA^{BA}**] = 20%). Luminescence dissymmetry factor (g_{lum}): ~1.0 × 10^{-2}. Source data are provided as a Source Data file.

3. Supplementary Tables

Supplementary Table 1 | Gelation properties of BTA^{BA} (5.3 mM) in various solvents at 25 °C

Solvent	Phase
Water	Insoluble
Acetonitrile	Insoluble
Methanol	Insoluble
Ethanol	Insoluble
Acetone	Insoluble
Dioxane	Insoluble
THF	Insoluble
$CHCl_3$	Insoluble
Ethyl acetate	Insoluble
CH_2Cl_2	Insoluble
Toluene	Insoluble
Cyclohexane	Insoluble
Hexane	Insoluble
DMF	Solution
DMF/water (7/3, v/v)	Gel

Supplementary Table 2 | Gelation properties of BTA^{BA} (5.3 mM) in DMF/water at different solvent compositions (volume ratios) at 25 °C

Volume ratio	Phase
10/0	Solution
9/1	Solution
8/2	Solution
7/3	Gel
6/4	Gel
5/5	Gel
4/6	Gel
3/7	Gel
2/8	Partial Gel
1/9	Partial Gel
0/10	Insoluble

Supplementary Table 3 | Statistical *ee* values of the endo isomers obtained from a total of 76 runs of the Diels–Alder reaction catalyzed at 25 °C by 76 different batches of (–)-PBTA^{BA}/Cu^{2+} or (+)-PBTA^{BA}/Cu^{2+} at [Cu^{2+}]/[BTA^{BA}] = 1.0% in DMF/water (7/3, v/v; 1 mL)

Entry	Ligand	% *ee*	Entry	Ligand	% *ee*	Entry	Ligand	% *ee*
1	(–)-**PBTA^{BA}**	18.9 (*S*)	27	(–)-**PBTA^{BA}**	16.6 (*S*)	53	(–)-**PBTA^{BA}**	16.9 (*S*)
2	(+)-**PBTA^{BA}**	17.5 (*R*)	28	(+)-**PBTA^{BA}**	17.7 (*R*)	54	(–)-**PBTA^{BA}**	21.4 (*S*)
3	(–)-**PBTA^{BA}**	16.1 (*S*)	29	(–)-**PBTA^{BA}**	18.5 (*S*)	55	(+)-**PBTA^{BA}**	22.5 (*R*)
4	(+)-**PBTA^{BA}**	17.0 (*R*)	30	(+)-**PBTA^{BA}**	18.0 (*R*)	56	(+)-**PBTA^{BA}**	17.7 (*R*)
5	(–)-**PBTA^{BA}**	17.5 (*S*)	31	(+)-**PBTA^{BA}**	18.9 (*R*)	57	(–)-**PBTA^{BA}**	23.3 (*S*)
6	(+)-**PBTA^{BA}**	17.5 (*R*)	32	(–)-**PBTA^{BA}**	19.8 (*S*)	58	(+)-**PBTA^{BA}**	20.7 (*R*)
7	(+)-**PBTA^{BA}**	21.0 (*R*)	33	(–)-**PBTA^{BA}**	23.7 (*S*)	59	(+)-**PBTA^{BA}**	21.5 (*R*)
8	(–)-**PBTA^{BA}**	19.4 (*S*)	34	(+)-**PBTA^{BA}**	18.9 (*R*)	60	(–)-**PBTA^{BA}**	21.8 (*S*)
9	(+)-**PBTA^{BA}**	18.2 (*R*)	35	(–)-**PBTA^{BA}**	20.9 (*S*)	61	(+)-**PBTA^{BA}**	23.7 (*R*)
10	(–)-**PBTA^{BA}**	17.0 (*S*)	36	(+)-**PBTA^{BA}**	18.4 (*R*)	62	(–)-**PBTA^{BA}**	13.1 (*S*)
11	(–)-**PBTA^{BA}**	14.2 (*S*)	37	(+)-**PBTA^{BA}**	20.9 (*R*)	63	(+)-**PBTA^{BA}**	10.9 (*R*)
12	(+)-**PBTA^{BA}**	12.8 (*R*)	38	(–)-**PBTA^{BA}**	17.9 (*S*)	64	(–)-**PBTA^{BA}**	24.7 (*S*)
13	(+)-**PBTA^{BA}**	17.2 (*R*)	39	(–)-**PBTA^{BA}**	13.9 (*S*)	65	(–)-**PBTA^{BA}**	15.2 (*S*)
14	(–)-**PBTA^{BA}**	22.8 (*S*)	40	(+)-**PBTA^{BA}**	13.7 (*R*)	66	(+)-**PBTA^{BA}**	18.7 (*R*)
15	(+)-**PBTA^{BA}**	17.4 (*R*)	41	(–)-**PBTA^{BA}**	19.6 (*S*)	67	(+)-**PBTA^{BA}**	17.4 (*R*)
16	(–)-**PBTA^{BA}**	18.6 (*S*)	42	(–)-**PBTA^{BA}**	24.8 (*S*)	68	(–)-**PBTA^{BA}**	12.7 (*S*)
17	(–)-**PBTA^{BA}**	18.7 (*S*)	43	(+)-**PBTA^{BA}**	17.0 (*R*)	69	(–)-**PBTA^{BA}**	20.6 (*S*)
18	(+)-**PBTA^{BA}**	19.0 (*R*)	44	(+)-**PBTA^{BA}**	12.6 (*R*)	70	(+)-**PBTA^{BA}**	14.0 (*R*)
19	(+)-**PBTA^{BA}**	15.0 (*R*)	45	(–)-**PBTA^{BA}**	11.4 (*S*)	71	(+)-**PBTA^{BA}**	14.5 (*R*)
20	(–)-**PBTA^{BA}**	21.7 (*S*)	46	(+)-**PBTA^{BA}**	15.8 (*R*)	72	(–)-**PBTA^{BA}**	9.0 (*S*)
21	(+)-**PBTA^{BA}**	18.5 (*R*)	47	(+)-**PBTA^{BA}**	17.3 (*R*)	73	(+)-**PBTA^{BA}**	17.2 (*R*)
22	(–)-**PBTA^{BA}**	13.6 (*S*)	48	(+)-**PBTA^{BA}**	17.5 (*R*)	74	(–)-**PBTA^{BA}**	15.6 (*S*)
23	(+)-**PBTA^{BA}**	13.0 (*R*)	49	(–)-**PBTA^{BA}**	19.4 (*S*)	75	(–)-**PBTA^{BA}**	21.3 (*S*)
24	(–)-**PBTA^{BA}**	12.7 (*S*)	50	(+)-**PBTA^{BA}**	18.7 (*R*)	76	(–)-**PBTA^{BA}**	20.8 (*S*)
25	(+)-**PBTA^{BA}**	16.0 (*R*)	51	(–)-**PBTA^{BA}**	23.9 (*S*)			
26	(–)-**PBTA^{BA}**	16.9 (*S*)	52	(+)-**PBTA^{BA}**	19.1 (*R*)			

With (–)-**PBTA^{BA}** and (+)-**PBTA^{BA}**, the average *ee* values were 18.3 ± 3.9 (*S*) and 17.5 ± 2.8 (*R*), respectively.

Supplementary Table 4 | *ee* values of the endo isomers obtained from the Diels–Alder reaction catalyzed at 25 °C by a single (+)-PBTA^{BA}/Cu^{2+} sample at [Cu^{2+}]/[BTA^{BA}] = 1.0% recycled by centrifugation and redispersion in DMF/water (7/3, v/v; 1 mL)

Recycle	% *ee*
0	13.9 (*R*)
1	10.6 (*R*)
2	8.9 (*R*)
3	2.5 (*R*)

4. Supplementary References

1. Jin Q. X. *et al*. Self-assembly of copper(II) ion-mediated nanotube and its supramolecular chiral catalytic behavior. *Langmuir* **27**, 13847–13853 (2011).
