# Supplementary material for: Asymmetric catalysis mediated by a mirror symmetry-broken helical nanoribbon
Source: Nat Commun. 2019 Sep 4;10:3976. doi: 10.1038/s41467-019-11840-3 (PMC6726595; doi:10.1038/s41467-019-11840-3)
Supplement: Supplementary file 2 — Peer Review [file 41467_2019_11840_MOESM2_ESM.pdf]

Reviewer #1 (Remarks to the Author):

Title: Asymmetric catalysis mediated by a chiral symmetry-broken helical nanoribbon
Authors: Shen et al.

The authors have a unique supramolecular system based on an achiral benzene-1,3,5-tricarboxamise that demonstrates both mirror symmetry breaking AND chiral amplification at the supramolecular level. Supported with careful and exhaustive and well-described experiments. Most importantly, they also demonstrate that these chiral fibers, when complexed with Cu2+, can catalyze the enatioselective Diels-Alder reaction between aza-chalcone and cyclopentadiene. Once again, the authors demonstrate this with extensive experiments and all the proper control reactions. This is extremely nice original work very worthy of publication in Nature Communications and will definitely be of interest to an audience with broad research interests.

Corrections and suggested changes:

The authors should better stress and distinguish mirror symmetry breaking (stochastic) from chiral amplification (via secondary nucleation). (perhaps in the title too?)

Page 2 line 4: In rare cases, their absolute chirality is biased by mirror symmetry breaking.

Throughout the document: Replace 'polymeric assemblies' and 'polymer' with '"supramolecular polymer assemblies' or 'supramolecular assemblies'.

Page 2 line 18: Our achievement MAY provide a fundamental breakthrough…

Page 3 line 3: Which has attracted long-term attention…

Page 6 line 22 – …repeated cycles of secondary nucleation. This is analogous to chiral amplification via thermal cycling or attrition in conglomerate crystal systems - ADD REFERENCE.

Page 9: replace 'certainly revolutionizes' with 'may revolutionize'

Reviewer #2 (Remarks to the Author):

This manuscript addresses one approach for producing active molecules from a mixture of achiral motifs as catalyst for Diels-Alder reaction. The topic is adequate for this journal as it fits within its aim & scope and will be useful to its wide readership. Although the reported research builds upon previous reports from the authors, this manuscript describes a new application for these materials with impact on asymmetric catalysis.
The research is quite well conducted, in what concerns the catalytic performance, although some points needs to be clarified. As such, some aspects of the manuscript deserve revision by the authors as follows:
1. Concerning the obtained catalytic results, authors have conducted several experiments to optimize the catalytic conditions to achieve enantioselective results for the desired product.
Most probably is possible to recycle the catalyst, taking this into account it was not clear from results in Fig. 3d whether the several runs were from a single catalyst sample recycled by separation/reheating/cooling under stirring and running again or from different batch synthesis. Nevertheless, recycling experiments should be conducted for several catalytic recycling runs to show how the catalyst behaves.

2. After a catalytic reaction the catalysts present the same properties and structure found at for the fresh catalyst? Authors should demonstrate this as well.
3. In each batch of the synthesis of the catalysts they always show the same statistics for asymmetric induction being dependent on the stirring rate for example. From data in Fig. 3d or in Suppl. Table 3, it would be interesting for authors to demonstrate what would be the average (or median values) along with the variance found for each of the (+) and (-) catalysts in terms of ee. In addition, it would be interesting to correlate each value with that found for the CD of each catalyst (as in Suppl. Fig. 20).

Overall this work demonstrates a good application of a concept that is relevant, which finds similarities in other materials (e.g. Y. Han, L. Zhao, J.Y. Ying, Adv. Mater. 19 (2007) 2454; and other papers that followed this one on catalytic applications). Therefore, this manuscript can be accepted after these points have been revised by the authors.

Reviewer #3 (Remarks to the Author):

The manuscript "Asymmetric catalysis mediated by a chiral symmetry-broken helical nanoribbon" by Liu and co-workers reports a rather unique finding in which an achiral benzene-1,3,5-tricarboxamide-based molecule assembles into a chiral superstructure and the sample preparation treatment allows to induce an excess helicity, either (+) or (-) into the resulting suspensions. These were subsequently used in a Cu(II)-catalysed Diels-Alder reaction and it was observed that the sign of the suspension dictated the enantiomeric excess of the reaction product. Several helical supramolecular and polymeric systems have been shown to bias the outcome of an asymmetric reaction (Bouteiller, Suginome) but as far as I know symmetry breaking in an achiral starting material has not been employed to serve as a carrier for asymmetric catalysis. Ofcourse, the fact that symmetry breaking can result in high enantiomeric excesses in stirred crystallisations has been beautifully illustrated by Viedma/Vlieg/Noorduin/Blackmond and was recently also applied to several types of condensation reactions such as Mannich reactions and Michael additions (see S.B. Tsogoeva et al, Angew Chem. Int. Ed., 2009, 48, 590–594 and Chem. Eur. J., 2009,15, 10255–10262). Despite these earlier beautiful examples (many of which are not mentioned by the authors) this work is novel.

To comply with the high standards of Nat. Commun. This reviewer has a number of questions and proposed additions to increase the strength and impact of the current work.

1. Chiral symmetry breaking. The introduction is quite brief on previous examples on chiral symmetry breaking in stirred crystallisations (Viedma ripening or attrition enhance deracemizations), and the use of helical scaffolds (covalent or noncovalent) in asymmetric synthesis. Also the selected examples seem to be randomly picked rather that the outcome of a thorough literature study. I suggest the authors reread the seminal papers in this area and select examples more systematically rather than randomly. I really miss the outstanding contributions of Suginome and Blackmond in this field.

2. The gel system. The authors mention a complicated but reproducible preparation procedure to obtain optically active "suspensions" from the achiral BTA. I have two issues here.
(a) Issue one is the use of the achiral triacid. This molecule has been reported by the group of Schmidt in 2012 (Soft Matter, 2012, 8, 66) as an excellent gelator in aqueous media that becomes fluorescent upon gel formation. This paper is unfortunately nowhere mentioned and it seems that the authors missed it. I strongly suggest that credit is given to this original work, which for some part is repeated by these authors in the Supplementary Info (ie the fluorescence experiments, pH sensitivity and the gelation experiments with microscopy work).
(b) The second issue is with the suspension formation after cooling, which is optically active. First of all, a suspension is not necessarily a gel as it lack viscoelasticity (sometimes some confusion arised with this reviewer). Secondly, what constitutes this suspension? Is it microcrystallites? Was this checked with polarised optical microscopy? These microcrystallites can also immobilise a solvent

because the large surface area of the crystals stick together strongly. And if these are microcrystals, what does CD actually measure?? I was under the impression that CD measures whatever is in solution, not the white precipitate. In addition, the presence of such a white suspension should lead to a large amount of scattering. What is still in solution and why is there no scattering?? And why are the CD effects (and gabs) so extremely large? For a normal BTA the molar circular dichroism is around 40 L/mol.cm. In this case, the optical path length is nowhere given so it is impossible to calculate the molar circular dichroism (De = CD effect / (cxlx32980). The values here seem to be much larger judging from Gabs. This is strange. Please calculate values for molar circular dichroism, give optical path lengths and explain in more detail what the CD actually represents. It is now unclear.

3. Helical nanoribbons. It is unexplained how the molecular packing of the BTAs are within the helical nanoribbons (that compose the optically active suspensions) and the authors are not very descriptive on this issue. In the Soft Matter paper it is suggested that a helical aggregate stabilised by intermolecular H-bonds is present. I actually doubt that. Given the IR data, the high fluorescence in the aggregated state and the large changes in fluorescence when diluting the system, it seems that a more planar intercalation of BTAs is present that form a sheet and than roll up in helical ribbons. Could the authors find more arguments on which type of organisation of the BTAs is present in the nanoribbons, and share them with the reader? In addition, this has large consequences for the catalysis because it may explain why the ee's are rather low in this system.

4. Catalysis.
(a) The authors select the Cu(II) catalysed Diels Alder as their reaction of choice. Why this choice? And where are the relevant references. Roelfes and Engbertsen did a lot of work on this reaction in chiral environments (proteins, DNA) but the only reference to Roelfes is for a hydration of enones. I really urge the authors to add relevant references and explain why this system is selected. I guess this system is selected as it is supersensitive for a chiral environment as shown by Roelfes in a number of contributions. So it would be the easiest catalytic system to test this catalysis. Now it seems randomly picked which undoubtedly it was not. Please elaborate on the choice of the catalytic system. In addition, I always like comparisons and it seems that the transfer of the chirality (although superamazing) is rather modest (ee = 46%). Upon intercalating Cu(II) complexes into DNA superhigh ee's were typically obtained. Presumably it means that the Cu(II) is not always properly positioned close to a chiral environment.
(b) How do the catalysis experiment look like? Does the suspensions remain a suspension or do they observe changes because of the formation of apolar products? And how was stirring done? In these heterogeneous mixtures proper stirring is often an issue and crucial for the success of the catalytic reaction. A bit more explanations need to be added to the ESI on how exactly the catalytic experiments were done.

I very much like this work and I think it could become suitable for the high standards of Nat Commun. But I also think more details, as described above, are required and a better reference section are needed in order to make this manuscript suitable for acceptance.

Point-to-Point Answers to Reviewers' Comments

For Reviewer 1

[1] The authors have a unique supramolecular system based on an achiral benzene-1,3,5-tricarboxamise that demonstrates both mirror symmetry breaking AND chiral amplification at the supramolecular level. Supported with careful and exhaustive and well-described experiments. Most importantly, they also demonstrate that these chiral fibers, when complexed with Cu^{2+}, can catalyze the enantioselective Diels-Alder reaction between aza-chalcone and cyclopentadiene. Once again, the authors demonstrate this with extensive experiments and all the proper control reactions. This is extremely nice original work very worthy of publication in Nature Communications and will definitely be of interest to an audience with broad research interests.

=> We appreciate this highly encouraging remark.

[2] The authors should better stress and distinguish mirror symmetry breaking (stochastic) from chiral amplification (via secondary nucleation). (perhaps in the title too?)

=> Please pay attention to the description in the original manuscript, on page 7, lines 6–14, where we clearly described that both chiral symmetry breaking and subsequent chiral amplification are essential for the formation of (*P*)- or (*M*)-dominant helical nanoribbons from achiral $\mathbf{BTA}^{\mathbf{BA}}$.

[3] The authors Page 2 line 4: In rare cases, their absolute chirality is biased by mirror symmetry breaking.

Throughout the document: Replace 'polymeric assemblies' and 'polymer' with 'supramolecular polymer assemblies' or 'supramolecular assemblies'.

Page 2 line 18: Our achievement MAY provide a fundamental breakthrough…

Page 3 line 3: Which has attracted long-term attention…

Page 9: replace 'certainly revolutionizes' with 'may revolutionize'.

=> According to the reviewer's suggestions, we revised the corresponding parts of the manuscript.

[4] Page 6 line 22 – …repeated cycles of secondary nucleation. This is analogous to chiral amplification via thermal cycling or attrition in conglomerate crystal systems - ADD REFERENCE

=> We added the following sentence to the revised manuscript, on page 7, lines 13–14: "as proposed for the mechanism of chiral amplification via thermal cycling or attrition in conglomerate crystal

systems[9–11]". Accordingly, we newly added references 9–11 describing grinding-induced chiral amplification of crystals.

For Reviewer 2

[1] This manuscript addresses one approach for producing active molecules from a mixture of achiral motifs as catalyst for Diels-Alder reaction. The topic is adequate for this journal as it fits within its aim & scope and will be useful to its wide readership. Although the reported research builds upon previous reports from the authors, this manuscript describes a new application for these materials with impact on asymmetric catalysis. The research is quite well conducted, in what concerns the catalytic performance, although some points needs to be clarified.

=> We appreciate this encouraging remark.

[2] Concerning the obtained catalytic results, authors have conducted several experiments to optimize the catalytic conditions to achieve enantioselective results for the desired product. Most probably is possible to recycle the catalyst, taking this into account it was not clear from results in Fig. 3d whether the several runs were from a single catalyst sample recycled by separation/reheating/cooling under stirring and running again or from different batch synthesis. Nevertheless, recycling experiments should be conducted for several catalytic recycling runs to show how the catalyst behaves.

=> In Fig. 3d, the catalyst was prepared for each run of the Diels-Alder reaction. To avoid this confusion, we added a short note to the corresponding parts in the revised manuscript and revised Supplementary Information.

=> According to the reviewer's suggestion, we newly conducted the Diels-Alder reaction using a catalyst recycled by centrifugation/redispersion and added the results to revised Supplementary Table 4. The *ee* value decreased when the catalytic reaction was performed using a recycled catalyst.

[3] After a catalytic reaction the catalysts present the same properties and structure found at for the fresh catalyst? Authors should demonstrate this as well.

=> According to the reviewer's suggestion, we newly conducted SEM for a dried catalytic reaction mixture and added the results to revised Supplementary Fig. 26. In SEM, shorter nanoribbons than those before the catalytic reaction were observed.

[4] In each batch of the synthesis of the catalysts they always show the same statistics for asymmetric induction being dependent on the stirring rate for example. From data in Fig. 3d or in Suppl. Table 3, it would be interesting for authors to demonstrate what would be the average (or median values) along with the variance found for each of the (+) and (-) catalysts in terms of *ee*.

=> We appreciate this suggestion. We calculated the average *ee* values with the variance from the *ee* values in Supplementary Table 3. With (–)-**PBTA**$^{\mathbf{BA}}$ and (+)-**PBTA**$^{\mathbf{BA}}$, the average *ee* values were 18.3 ± 3.9 (*S*) and 17.5 ± 2.8 (*R*), respectively.

[5] In addition, it would be interesting to correlate each value with that found for the CD of each catalyst (as in Suppl. Fig. 20).

=> We investigated the relationship between the *ee* values of the endo isomers and the CD intensities of the corresponding **PBTA**$^{\mathbf{BA}}$ catalysts as shown in newly added Supplementary Fig. 25 but did not find any correlation.

For Reviewer 3

[1] The manuscript "Asymmetric catalysis mediated by a chiral symmetry-broken helical nanoribbon" by Liu and co-workers reports a rather unique finding in which an achiral benzene-1,3,5-tricarboxamide-based molecule assembles into a chiral superstructure and the sample preparation treatment allows to induce an excess helicity, either (+) or (-) into the resulting suspensions. These were subsequently used in a Cu(II)-catalysed Diels-Alder reaction and it was observed that the sign of the suspension dictated the enantiomeric excess of the reaction product. Several helical supramolecular and polymeric systems have been shown to bias the outcome of an asymmetric reaction (Bouteiller, Suginome) but as far as I know symmetry breaking in an achiral starting material has not been employed to serve as a carrier for asymmetric catalysis. Of course, the fact that symmetry breaking can result in high enantiomeric excesses in stirred crystallisations has been beautifully illustrated by Viedma/Vlieg/Noorduin/Blackmond and was recently also applied to several types of condensation reactions such as Mannich reactions and Michael additions (see S.B. Tsogoeva et al, Angew Chem. Int. Ed., 2009, 48, 590–594 and Chem. Eur. J., 2009,15, 10255–10262). Despite these earlier beautiful examples (many of which are not mentioned by the authors) this work is novel.

=> We appreciate this encouraging remark.

=> According to the reviewer's suggestion, we added suggested and related references to the revised manuscript.

[2] Chiral symmetry breaking. The introduction is quite brief on previous examples on chiral symmetry breaking in stirred crystallisations (Viedma ripening or attrition enhance deracemizations), and the use of helical scaffolds (covalent or noncovalent) in asymmetric synthesis. Also the selected examples seem to be randomly picked rather that the outcome of a thorough literature study. I suggest the authors reread the seminal papers in this area and select examples more systematically rather than randomly. I really miss the outstanding contributions of Suginome and Blackmond in this field.

=> We appreciate this suggestion. We added suggested and related references describing chiral symmetry breaking in crystalline systems and asymmetric synthesis using helical scaffolds to the revised manuscript.

[3] The gel system. The authors mention a complicated but reproducible preparation procedure to obtain optically active "suspensions" from the achiral BTA. I have two issues here.

(a) Issue one is the use of the achiral triacid. This molecule has been reported by the group of Schmidt in 2012 (Soft Matter, 2012, 8, 66) as an excellent gelator in aqueous media that becomes fluorescent upon gel formation. This paper is unfortunately nowhere mentioned and it seems that the authors missed it. I strongly suggest that credit is given to this original work, which for some part is repeated by these authors in the Supplementary Info (ie the fluorescence experiments, pH sensitivity and the gelation experiments with microscopy work).

=> We appreciate this suggestion. The solvent we used for the gelation of **BTA**$^{\mathbf{BA}}$ was DMF/water and therefore different from that used in the reported system. Nevertheless, we added the suggested reference, because we thought that such a reference is beneficial for the readers.

(b) The second issue is with the suspension formation after cooling, which is optically active. First of all, a suspension is not necessarily a gel as it lacks viscoelasticity (sometimes some confusion arose with this reviewer).

=> Please note that, as shown in Fig. 2, a gel forms when water is added to a DMF solution of **BTA**$^{\mathbf{BA}}$. Please also note that Fig. 2 clearly indicates that the preparation of the **BTA**$^{\mathbf{BA}}$ suspension from its gel requires stirring upon cooling after heating the gel to induce a gel-sol transition.

[4] What constitutes this suspension? Is it microcrystallites? Was this checked with polarised optical microscopy? These microcrystallites can also immobilise a solvent because the large surface area of the crystals stick together strongly.

=> Newly conducted polarized optical microscopy revealed that the suspension did not contain any crystalline fraction (Supplementary Fig. 17). Accordingly, XRD of a wet sample of **PBTA**$^{\mathbf{BA}}$ isolated by centrifugation displayed no diffraction peak (Supplementary Fig. 18).

[5] If these are microcrystals, what does CD actually measure?? I was under the impression that CD measures whatever is in solution, not the white precipitate. In addition, the presence of such a white suspension should lead to a large amount of scattering. What is still in solution and why is there no scattering?

=> Again, the suspension does not contain any crystalline fraction. For the absorption and CD measurements of the suspension or gel, we used a sandwich-type cuvette with a thickness of 0.1 mm in order to avoid light scattering (for example, Shinkai, S. *et al.* Thermal and light control of the sol-gel phase-transition in cholesterol-based organic gels. Novel helical aggregation modes as detected by circular-dichroism and electron-microscopic observation. *J. Am. Chem. Soc.* 1994, **116**, 6664–6676).

[6] Why are the CD effects (and g_{abs}) so extremely large? For a normal BTA the molar circular dichroism is around 40 L/mol.cm. In this case, the optical path length is nowhere given so it is impossible to calculate the molar circular dichroism (De = CD effect / (c × l × 32980). The values here seem to be much larger judging from G_{abs}. This is strange. Please calculate values for molar circular dichroism, give optical path lengths and explain in more detail what the CD actually represents. It is now unclear.

[7] Helical nanoribbons. It is unexplained how the molecular packing of the BTAs is within the helical nanoribbons (that compose the optically active suspensions) and the authors are not very descriptive on this issue. In the Soft Matter paper it is suggested that a helical aggregate stabilized by intermolecular H-bonds is present. I actually doubt that. Given the IR data, the high fluorescence in the aggregated state and the large changes in fluorescence when diluting the system, it seems that a more planar intercalation of BTAs is present that form a sheet and then roll up in helical ribbons. Could the authors find more arguments on which type of organization of the BTAs is present in the nanoribbons, and share them with the reader? In addition, this has large consequences for the catalysis because it may explain why the *ee* values are rather low in this system.

=> As described above, we used a sandwich-type cuvette with an optical path length of 0.1 mm to ensure a sufficient transmittance for the CD measurements. We added a short phrase to the Methods section of the revised manuscript as follows: "in sandwich-type quartz cuvettes with an optical path length of 0.1 mm".

=> The molar circular dichroism of **BTA^{BA}** in the helical nanoribbons was calculated as ~570 L/mol·cm. This value is much larger than those reported for chiral BTA derivatives. Note that **BTA^{BA}** carries three BA units at the periphery of the BTA core and therefore very special. Also note that some helicene nanoribbons, rather than helically twisted cofacial 1D stacks typical of ordinary BTA derivatives with amide-appended side chains, are known to show such amplified chiroptical behaviors (*JACS* **2018**, 140, 6235; $\Delta\varepsilon$ = ~800 L/mol·cm). In relation to this issue, we attempted to analyze by XRD a wet sample of **PBTA^{BA}** isolated by centrifugation, but did not find any diffraction (Supplementary Fig. 18), in consistent with the POM profile. As already described above, wet **PBTA^{BA}** is non-crystalline, and we were unable to obtain its structural characteristics. We would like to leave this issue to our future work. Please see the Discussion section, suggested by the editor.

=> Because the enantioselectivity of the reaction is not high, we assume, as suggested by this reviewer, that **BTA^{BA}** assembles into a rather planer, sheet structure, which rolls up to form a

helical nanoribbon, wherein the catalytic sites are likely located. Please also see the Discussion section, suggested by the editor.

[8] Catalysis.

The authors select the Cu(II) catalyzed Diels–Alder as their reaction of choice. Why this choice? And where are the relevant references. Roelfes and Engbertsen did a lot of work on this reaction in chiral environments (proteins, DNA) but the only reference to Roelfes is for a hydration of enones. I really urge the authors to add relevant references and explain why this system is selected. I guess this system is selected as it is supersensitive for a chiral environment as shown by Roelfes in a number of contributions. So it would be the easiest catalytic system to test this catalysis. Now it seems randomly picked which undoubtedly it was not. Please elaborate on the choice of the catalytic system.

[9] In addition, I always like comparisons and it seems that the transfer of the chirality (although superamazing) is rather modest (*ee* = 46%). Upon intercalating Cu(II) complexes into DNA superhigh *ee* values were typically obtained. Presumably it means that the Cu(II) is not always properly positioned close to a chiral environment.

=> We appreciate this reviewer's comment. As the reviewer described, the Cu^{2+}-catalyzed Diels–Alder cycloaddition reaction is highly sensitive to chiral environments as demonstrated by Roelfes and Engbertsen, and therefore we chose this reaction. We added to the revised manuscript a short description and the suggested references.

=> Your point that Cu(II) is not always properly positioned close to a chiral environment may be right. Nevertheless, as you also pointed out, we assume that modest enantioselectivity (*ee* = 46%) is due to the helical nanoribbon structure, which is formed by rolling up of a rather planer, sheet structure. The catalytic sites are located on such a gently curved surface, and therefore the enantioselectivity of the reaction is modest.

[10] How does the catalysis experiment look like? Do the suspensions remain a suspension or do they observe changes because of the formation of apolar products? And how was stirring done? In these heterogeneous mixtures proper stirring is often an issue and crucial for the success of the catalytic reaction. A bit more explanations need to be added to the ESI on how exactly the catalytic experiments were done.

=> We did not observe any apparent change during the catalytic reaction. The reaction mixture remained suspended.

=> According to the reviewer's suggestion, we added a short description of how we stirred the reaction mixture to the revised Supporting Information. The reaction mixture was stirred at

1200 rpm using an IKA® RCT Basic Magnetic Stirrer with a 5.0 × 10.0 mm Teflon-coated stirring bar.

REVIEWERS' COMMENTS:

Reviewer #1 (Remarks to the Author):

The authors have clearly addressed my comments and concerns in their revised manuscript. I look forward to seeing this very nice work published in Nature Communications.

Reviewer #2 (Remarks to the Author):

This work was very well conducted. Authors have answered all questions raised by the reviewers. It can therefore be published in this form.

Reviewer #3 (Remarks to the Author):

The revised version of the manuscript "Asymmetric catalysis mediated by a mirror symmetry-broken helical nanoribbon" by Aida and coworkers has taken all my previous comments into consideration. A significant amount of additional experiments was done and added to the revised manuscript. I find especially intriguing the formation of (+) and (-) suspensions, the graph in the ESI is really similar to the one published by Kundepudi on stirred crystallisations of NaClO, in which also a bimodal distribution of (+) and (-) enantiomorphs is formed. It is highly interesting that such behaviour is also found in suspension of supramolecular structures, and, more importantly, can be coupled to an enantioselective outcome of a asymmetric catalytic reaction. I have no reservations to recommend publication of this fascinating work.
